# Comparison of the protein composition of isolated extracellular vesicles from mouse brain and dissociated brain cell culture medium

**DOI:** 10.1371/journal.pone.0309716

**Published:** 2024-11-12

**Authors:** Zan Xu, Joshua Brian Foster, Rashelle Lashley, Xueqin Wang, Albert John Muhleman, Christopher Eli Masters, Chien-liang Glenn Lin

**Affiliations:** Department of Neuroscience, College of Medicine, The Ohio State University, Columbus, Ohio, United States of America; TotiCell Limited, Bangladesh, BANGLADESH

## Abstract

*Extracellular vesicles (EVs) play a crucial role in intercellular communication*. Characterizing EV protein composition is essential to understand EV function(s). Isolating EVs from cell culture medium is a common approach to study EVs, but it remains unclear whether EVs isolated from *in vitro* conditions accurately reflect physiological conditions of the same source *in vivo* tissues. Here, we analyzed the protein composition of EVs isolated from freshly dissected mouse forebrain and primary dissociated mouse forebrain culture medium. In total, 3,204 and 3,583 proteins were identified in EVs isolated *in vivo* and *in vitro*, respectively. Among the proteins identified from both EV sources, there was substantial overlap (~86%). While the overall proteome compositions were very similar, *in vitro* EVs were relatively enriched with transmembrane/GPI-anchored membrane and cytosolic proteins (MISEV2023 category 1 and 2) typically associated with EVs. Conversely, while both *in vivo* and *in vitro* EVs express likely non-EV proteins (MISEV2023 category 3), the *in vivo* samples were significantly more enriched with these probable contaminants, specifically ribosomal proteins. Our findings highlight that *in vitro* EVs may be representative of *in vivo* EVs when isolated from the same source tissue using similar methodology; however, each population of EVs have differences in both total and, primarily, relative protein expression likely due to differing levels of co-eluting contaminants. Therefore, these points must be considered when interpreting results of EV studies further suggesting that improved methods of isolation to reduce non-EV contaminants should be further investigated.

## Introduction

Extracellular vesicles (EVs) are small, membrane-bound particles that are secreted by all known cell types and carry a variety of biological molecules, including proteins, nucleic acids, and metabolites [1]. EVs (especially small EVs) play an important role in intercellular communication, immune regulation, and disease pathogenesis in the brain [2]. Identification of core and cargo EV protein components is important for understanding brain EV function. Currently, there is intense interest in developing liquid biopsies by characterizing the cargo of EVs released *in vivo* from patients to be used as biomarkers to track progression of disease and monitor treatment effect [3–9]. However, the composition of EVs can vary depending on the isolation method as no singular methods has been ascribed as the optimal method and, instead, various methods have been described to facilitate EV isolation [10–16]. Each of these methods individually has strengths and limitations in terms of isolating high yield, purified EVs. Therefore, it seems apparent that any one of these methods alone is unlikely to result in the isolation of a sufficiently purified EV population to reliably assess the expression of rare proteins that may be disease or treatment specific biomarkers.

In addition to the method of isolation, the source from which EVs are isolated is expected to change the protein composition. Isolation of EVs from cell culture medium has been a widely used approach in studies for characterization of EVs released from a specific- or limited, mixed- cell type [17–26]. This method has been commonly used to characterize cell-specific EV content and in understanding the basic functions of EVs from monoculture or coculture systems. Yet, it is not clear to what extent EVs isolated from these *in vitro* conditions reflect the physiological conditions of the original source tissue where the cells were isolated. Conversely, acute isolation of EVs from *in vivo* tissue may provide a more comprehensive understanding of the EV functions. However, isolation of EVs from *in vivo* tissue is susceptible to contamination from additional sources, such as intracellular vesicles or the vesicles that originate from cellular compartments, during the necessary disruption of tissue to acutely collect these EVs [2, 27]. While both *in vivo* and *in vitro* systems are used to assess EVs, to our knowledge, no other studies have directly compared the proteome content differences between EVs isolated from a common starting tissue source, especially of a neuronal source. Therefore, comparing the protein composition of EVs isolated from tissue and cell culture sourced from brain tissue directly is important to understand how comparable the two systems are for characterization and analysis of brain EVs.

In the current study, we aimed to compare the protein composition of EVs isolated from *in vivo* mouse forebrain and *in vitro* dissociated mixed neuronal forebrain primary coculture. The size exclusion chromatography (SEC) method was combined with ultrafiltration (UF) for the isolation of EVs. Mass spectrometry-based proteomics were used for the identification and quantification of proteins in isolated EVs. Our analysis revealed that ~86.4% of the identified proteins were common to both *in vitro* and *in vivo* EVs suggesting conservation of the EV proteomes. However, further analysis revealed vast differences in relative protein enrichment especially for proteins listed in categories 1, 2, and 3 defined by the minimal information for studies of extracellular vesicles 2023 (MISEV2023) guidelines [28] and the Vesiclepedia top 100 [29] commonly identified EV protein list. Our findings highlight that *in vitro* EVs may be representative of *in vivo* EVs when isolated from the same starting tissue source using a similar method; however, each population of EVs have clear differences in relative protein content that may be due to differential contamination sources and/or differences in EV composition.

## Materials and methods

### Ethics statement

This study was conducted in compliance with recommendations of the ARRIVE Guidelines (Animal Research: Reporting of In Vivo Experiments) for reporting *in vivo* experiments with research animals [30]. All experimental protocols in this study were approved by the Institutional Animal Care and Use Committee of The Ohio State University and followed the National Institutes of Health Guide for the Care and Use of Laboratory Animals. All experiments were performed in a manner that minimized animal number used, pain, and discomfort.

### Animals

For all *in vivo* experiments, we utilized 3-month-old wild-type FVB/NJ mice (Jackson Laboratories; Stock #001800). Four mice were used for the *in vivo* EV study. For *in vitro* studies, E16 embryos were dissected from pregnant FVB/NJ female dams and dissociated and incubated as described. Mice were housed on a 12-h light/dark cycle with access to food and water *ab libitum*.

### Mouse forebrain dissociation

Three-month old FVB/NJ mice were deeply anesthetized with isoflurane until non-responsive to toe pinch. Brains were removed quickly after cervical dislocation and placed in chilled artificial cerebrospinal fluid. The brains were placed and sliced in a brain matrix (Braintree scientific 1 mm, coronal). The forebrain sections were visually identified as those containing hippocampus (used to enrich cells from the cortex and hippocampus, while also including striatum and other subcortical structures, but excluding cerebellum) and were transferred to a 6-well plate with 75 U/ml of collagenase type 3 (Worthington Biochemical Corporation, Cat# NC9405360) in serum-free DMEM (Corning^™^, Cat# 10013CV). The tissue sections were subsequently finely chopped, and the tissue was incubated at 37°C for a total of 20 minutes with gentle shaking every 5 minutes. The tissue was further dissociated by pipetting. The dissociated tissue was spun at 300 × g for 5 min at 4°C to remove the undigested tissue as a pellet. The protein concentration of the supernatant was determined by DC protein assay kit II (Bio-Rad, Cat# 5000112) on an iMark Microplate Absorbance Reader (Bio-Rad, Cat# 1681130) to ensure an equal amount of starting tissue was used (3 mg) for each sample. The supernatant was transferred to a fresh tube and spun at 2,000 × g for 10 min at 4°C to remove the cellular fraction. Next, the supernatant was spun at 10,000 × g for 30 min at 4°C to remove cell debris and large intracellular vesicles. Finally, to concentrate the samples, the supernatant was spun at 100,000 × g for 70 min in a Bechman (model Optima L-90K) ultracentrifuge and the pellets (P4, raw EVs) were resuspended in 500 μl 1× PBS for use as the input for SEC EV isolation. A schematic diagram of the procedure is presented in S1 Fig.

### Mouse primary dissociated forebrain cell cultures

Sixteen-day old embryos (E16) were harvested from pregnant dams. The pregnant dams were deeply anesthetized with isoflurane until unresponsive to toe pinch before cervical dislocation and subsequent cesarean section removal of the embryos. The forebrain (defined as the whole brain including cortex, hippocampus, striatum, and additional subcortical regions, but with the cerebellum removed) of each embryo was dissected in 1× PBS on ice. The brain tissue was mechanically dissociated in 1 mL of DMEM (Corning^™^, Cat# 10013CV) containing papain (20 U/ml, Sigma Aldrich, Cat# P3125) and was incubated for 30 min at 37°C. The dissociated cells were then separated from the remaining undigested tissue by a 70 μm cell strainer (MTC Bio, Cat# C4070). Dissociated cells were added to 14 mL of plating media (DMEM supplemented with 5% fetal bovine serum [FBS, Alkali Scientific, Cat# FB72]) and plated into T75 flasks (VWR, Cat# 10062–860). Embryos from 2 dams were combined to generate 4 flasks of cells (each flask representing 1 replicate). At the end of day *in vitro* (DIV) 1, the plating media was fully replaced with fresh maintenance media (DMEM supplemented with 0.5% FBS, and 1× B27 [Invitrogen, Cat# 17504044]). The percentage of supplemented FBS was reduced by 90% to prevent glial proliferation. Beginning on DIV2 and continuing on every third day during DIV 2–14, 50% of the medium was replaced with fresh maintenance media also containing 100 μg/ml penicillin-streptomycin (Gibco, Cat# 15140122). On DIV 15, the maintenance media was fully removed, the cells were gently washed with 1× PBS three times to remove accumulated EVs and cellular debris. Extracellular vesicle-free, FBS-free DMEM was added to the flasks. On DIV 16, after allowing for EV accumulation in the DMEM for 24 hr, the media was collected and spun down at 1,000 × g for 10 min to remove the large debris and dead cells. The supernatant was collected and further spun at 10,000 × g for 30 min to remove large intracellular vesicles and additional cellular debris. The supernatant was then concentrated (and initially purified) down to 500 μl using UF (Amicon Ultra-15, 100 kDa membrane, Cat# UFC910024) and used as input for EV isolation via SEC. The 100 kDa membrane is predicted to trap vesicles ≥10 nm for collection. A schematic diagram of the procedure is presented in S1 Fig.

### Izon SEC column EV isolation and UF concentration

The raw EVs were isolated from both dissociated *in vivo* adult brain and embryonic *in vitro* neuron-astrocyte culture medium using a qEV SEC column (Izon) following the manufacture’s manual. The qEVOriginal / 35 nm column (Izon, Cat# ICO-35), designed for separating EVs between 35–350 nm from a 500 μL biological sample, was used for this study. Briefly, qEVOriginal columns were flushed with 1× PBS before the samples were loaded. All the samples were concentrated into 500 μl in 1× PBS as described above before being loaded onto the column. The qEV Automatic Fraction Collector (AFC, Izon, Cat# AFC-V2) was programmed for automatic isolation of the EVs. A total of 4 fractions (corresponding to fraction 7–10; fractions 1–6 correspond to the void volume that is deplete of EVs) were collected for each sample. Fraction 9 and fraction 10, which contained the small EVs enriched in sizes ranging between 50–150 nm, were pooled and concentrated (as well as further purified) by UF (Amicon Ultra-4, 100 kDa membrane, Cat# UFC810096) to a final volume of 50 μl. The 100 kDa membrane for UF was selected as the pore size is predicted to isolate EVs of 10–20 nm and larger, but deplete smaller, empty vesicles and free protein under 100 kDa. A schematic diagram of the procedure is presented in S1 Fig. The obtained samples were subjected to Western blot analysis to assess markers of EVs, including Tsg 101, Alix and CD81. Results are presented in S2 Fig.

### Nanoparticle tracking analysis

The concentration and size of the vesicles were characterized by Nanoparticle Tracking Analysis (NTA; Malvern Pharmaceuticals, NanoSight NS300) using the manufacturer’s instructions. A small portion of the isolated EVs were diluted 1:1000 with 1× PBS to achieve 15 to 50 particles per frame for tracking. The sample was injected via syringe and slowly pumped into the chamber for analysis. A laser passed through the chamber and the particles in suspension in the path of the beam scattered the light which could be visualized via 20× magnification. Each sample was analyzed three times for 30s in the chamber to generate an aggregate average.

### Sample clean-up and protein digestion via S-Trap

After NTA analysis, the remaining samples were denatured by the addition of 5× SDS loading buffer (250 mM Tris—HCl pH 6.8, 50% glycerol, 10% SDS, 500 mM DTT, 0.5% bromophenol blue, 5% β-mercaptoethanol) to a final concentration of 1× and heated at 95°C for 5 min. Up to 50 ug of the samples were used for trypsin digestion. The samples were purified of macromolecules other than proteins using Suspension Trapping (S-Trap) micro columns (≤ 100 μg) per the manufacturer’s instructions (Protifi, Cat# K02MICRO10). Briefly, 5 μL of 50 mM ammonium bicarbonate containing 5 μg/μL DTT was added, and the sample was incubated at 65°C for 15 min followed by addition of 5 μL of 50 mM ammonium bicarbonate containing 15 μg/μL iodoacetamide (incubated at RT for 15 min in the dark). Samples were then acidified by adding 12% phosphoric acid (1:10 v/v acid to sample). For every 25 μL of sample, 165 μL of Triethylammonium bicarbonate (TEAB 1M)/MeOH (10:90 v/v) was added and then loaded to the S-Trap spin column for further washing. Samples were centrifuged at 4,000 × g for 3 min (4°C) to remove supernatant. Then, 150 μL of TEAB/MeOH (10:90 v/v) was added to the spin column as wash solution and the trap was washed 3–6 times dependent upon the initial loading volume. After the final wash, sequencing grade trypsin dissolved in 50 mM TEAB was added and digested O/N at 37 °C. The following day, peptides were eluted by adding 40 μL of 50 mM TEAB, 0.1% formic acid (FA) and 0.1% FA in acetonitrile (50:50), sequentially. The sample was pooled together and dried in a vacufuge and resuspended in 20 μL of 50 mM acetic acid. Peptide concentration was determined by nanodrop (A280nm). The samples were then submitted for mass spectrometry analysis.

### Mass spectrometry

Experimental procedures were performed similar as previously indicated with modifications [31]. Capillary-liquid chromatography-nanospray tandem mass spectrometry (Capillary-LC/MS/MS) was performed on an Orbitrap Fusion mass spectrometer (Thermo Scientific, Cat# FETD2-10002) equipped with an EASY-Spray^™^ Source operated in positive ion mode. Samples were separated on an EASY-Spray^™^ nano column (PepMap^™^ RSLC, C18 3μ 100A, 75μm X150mm Thermo Scientific) using a 2D rapid separation liquid chromatography HPLC system from Thermo Scientific. Each sample was injected into the μ-Precolumn Cartridge (Thermo Scientific) and desalted with 0.1% FA in water for 5 minutes. The injector port was then switched to inject, and the peptides were eluted off of the trap onto the column. Mobile phase A was 0.1% FA in water while acetonitrile (with 0.1% FA) was used as mobile phase B. Flow rate was set at 300nL/min. Mobile phase B was increased from 2% to 16% in 105 min and then increased from 16–25% in 10 min and again from 25–85% in 1 min and then kept at 95% for another 4 min before being brought back quickly to 2% in 1 min. The column was equilibrated at 2% of mobile phase B (or 98% A) for 15 min before the next sample injection.

MS/MS data was acquired with a spray voltage of 1.6 kV and a capillary temperature of 305°C was used. The scan sequence of the mass spectrometer was based on the preview mode data dependent TopSpeed^™^ method. The analysis was programmed for a full scan recorded between m/z 375–1500 and a MS/MS scan to generate product ion spectra to determine amino acid sequence in consecutive scans starting from the most abundant peaks in the spectrum in the next 3 seconds. To achieve high mass accuracy MS determination, the full scan was performed at Fourier transformation (FT) mode and the resolution was set at 120,000 with internal mass calibration. The automatic gain control (AGC) target ion number for FT full scan was set at 4 × 105 ions, maximum ion injection time was set at 50 ms and micro scan number was set at 1. Multistage mass spectrometry (MSn) was performed using higher energy collisional dissociation (HCD) in ion trap mode to ensure the highest signal intensity of MSn spectra. The HCD collision energy was set at 32%. The AGC target ion number for ion trap MSn scan was set at 30,000 ions, maximum ion injection time was set at 35 ms and micro scan number was set at 1. Dynamic exclusion is enabled with a repeat count of 1 within 60s and a low mass width and high mass width of 10ppm.

Data were searched using Mascot Daemon by Matrix Science version 2.7.0 (Boston, MA) via Proteome Discoverer (version 2.4, Thermo Scientific) and the database searched against the most recent Uniprot databases. The mass accuracy of the precursor ions was set to 10 ppm, accidental pick of one ^13^C peaks was also included into the search. The fragment mass tolerance was set to 0.5 Da. Carbamidomethylation (Cys) is used as a fixed modification and considered variable modifications were oxidation (Met) and deamidation (N and Q). Four missed cleavages for the enzyme were permitted. A decoy database was also searched to determine the false discovery rate (FDR) and peptides were filtered at 1% FDR. Proteins identified with at least two unique peptides were considered as reliable identification. Any modified peptides are manually checked for validation. Relative quantitation was done using the label free quantitation (LFQ) mass spectral peak intensity approach. Peptide precursor (MS1) intensities from the same protein were generated from ProteomeDiscoverer MASCOT search and summed up for quantitation comparison.

### Statistical analysis

The proteomic data for *in vivo* EVs and *in vitro* EVs were first merged and aligned (n = 4/group). Proteins that did not have values for at least 2 replicate samples for either *in vivo* or *in vitro* were excluded (4 proteins total were excluded). The proteins were analyzed using the Database for Annotation, Visualization and Integrated Discovery (DAVID) [32] to determine the cellular component (CC), biological process (BP), and molecular function (MF) of the proteins of the total proteome of both the *in vivo* and *in vitro* EVs. DAVID was also used to examine biological pathways enriched from proteins identified in both *in vivo* and *in vitro* samples through conducting a Kyoto Encyclopedia of Genes and Genomes (KEGG) analysis. The Benjamini—Hochberg method (p-value converted to -log_10_ for data presentation) was used to determine if a category within a gene ontology (GO) group or KEGG pathway is significantly enriched. For relative enrichment of individual proteins *in vivo* or *in vitro*, the LFQ for each protein was converted to a Z-score for comparison between sample types. Proteins enriched *in vivo*, enriched *in vitro*, or not enriched were analyzed by DAVID for CC, BP, MF (p < 0.05 presented) and KEGG pathway analysis (p < 0.05 for Benjamini-Hochberg test presented). Further, a list for each of the 5 categories from the MISEV2023 guidelines [28] were generated as well as the Vesiclepedia Top 100 proteins [29]. For the MISEV2023 categories, the HUGO Gene Nomenclature Committee website [33] was used to identify all proteins in the same family for those marked as having multiple proteins. All proteins from both sample types were cross-referenced with these lists and heatmaps (generated online by Heatmapper [34]) were generated from the replicate LFQ Z-scores for each sample type. The proteins from each categorical list were further analyzed for significant enrichment to a sample type (*in vivo* or *in vitro*) using a Students T-test comparing the Z-scores from the 4 replicates for each of the *in vivo* and *in vitro* EV sample types. The statistical values presented are indicated in each figure but represent at least p < 0.05.

## Results and discussion

### Biophysical characterization of EVs isolated from mouse intact brain and dissociated culture

**Fig 1:**
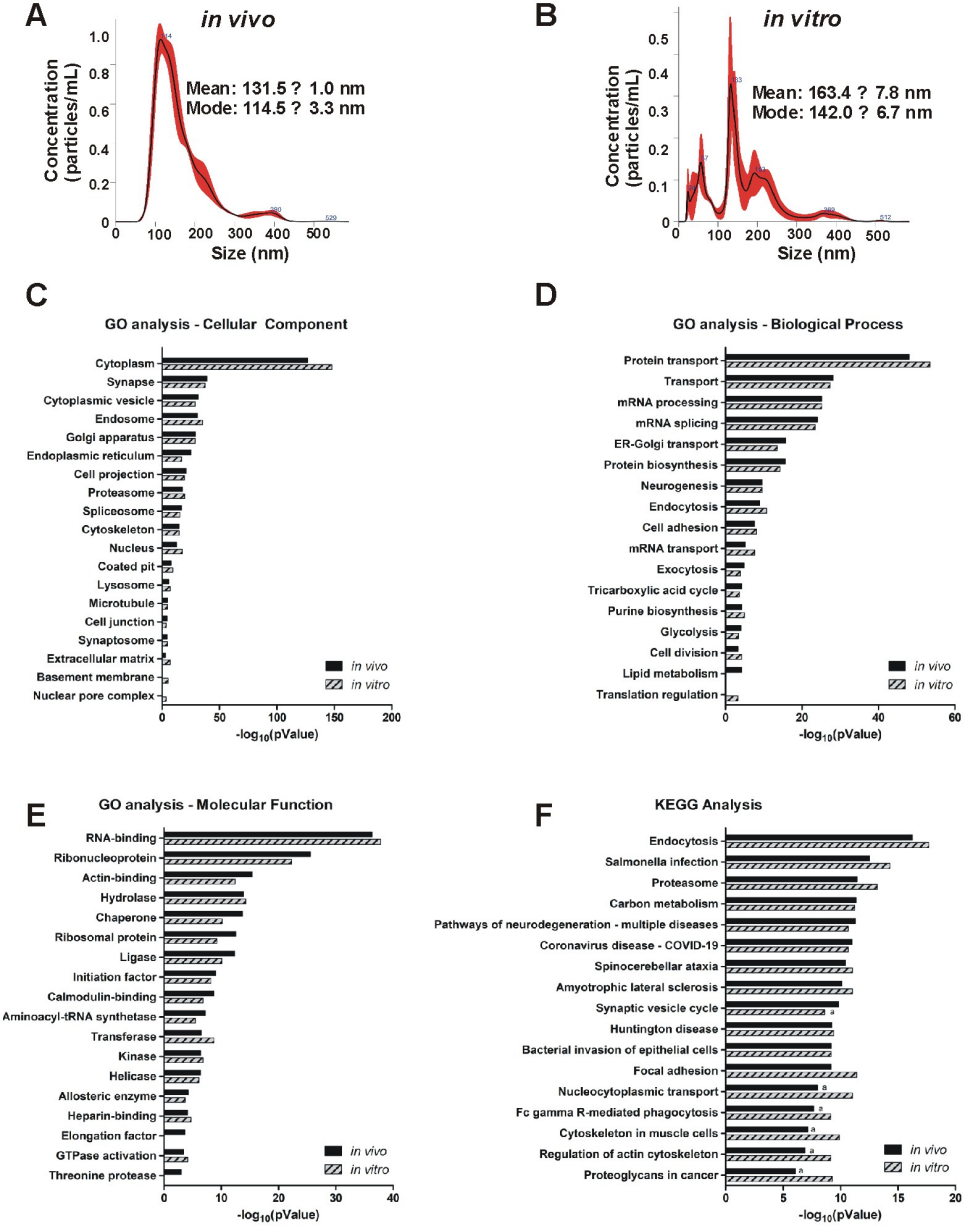
Characterization of EVs isolated via SEC and UF from mouse forebrain and primary culture. EVs were collected from either (1) acutely dissociated mouse forebrain, *in vivo* EVs, or (2) primary cells dissociated from embryonic mouse forebrain, *in vitro* EVs. **(A, B)** We first characterized the biophysical properties of both EV groups using NTA. **(A)** *In vivo* EVs yielded high density EVs with an average diameter of 131.5 nm and a peak at 114.5 nm while **(B)** *in vitro* EVs yielded lower concentrations as well as slightly larger (mean: 163.4 nm; primary peak: 142.0 nm) EVs. For both EV groups, the majority of EVs assessed fell within the typical parameters for small, exosome-like EVs (30–200 nm). **(C-E)** The protein content of each EV group was assessed using GO analysis of mass spectrometry data. A total of 3,204 and 3,583 proteins were identified in *in vivo* and *in vitro* EVs, respectively, and used for analysis. **(C)** Principle CC analysis of both *in vivo* and *in vitro* EVs yielded very similar term enrichment, specifically with the top terms cytoplasm and synapse followed by cytoplasmic vesicle, endosome, and Golgi apparatus consistently being the top 5 most significantly enriched terms. **(D)** Consistently, principle BP analysis shows that the same top 4 terms (in the same order) are enriched for both *in vivo* and *in vitro* including: protein transport, transport, mRNA processing, and mRNA splicing. **(E)** Principle MF analysis, again, suggests comparable term enrichment between the EV groups with the top terms being RNA-binding and ribonucleoprotein, followed by actin-binding, hydrolase, and chaperone. (**F**) KEGG pathway enrichment analysis indicates similar pathway enrichment between *in vivo* and *in vitro* EVs, with the most enriched pathways being endocytosis, salmonella infection, proteasome, and focal adhesion. Data displayed is the Benjamini-Hochberg p-value for term enrichment converted to -log_10_. Data shown in **C-E** is p < 0.001. Data shown in **F** is the Benjamini-Hochberg p < 1e^-9^. Values denoted in **F** with “a” were found to be present, but with a Benjamini-Hochberg p ≥ 1e^-9^. n = 4 replicates per EV group.

A number of studies have reported the procedures for the isolation of EVs from brain tissues [2, 35]. The protocol generally starts with a gentle dissociation of the tissue to free EVs from the extracellular space followed by sequential centrifugations (in a series of increasing speed) to remove cells, cellular debris, microvesicles, non-EV vesicles, and free protein. The EVs are then isolated using one of various approaches including, density/gradient centrifugation, UF, immunoprecipitation, or SEC to obtain exosome-enriched EVs [36–38]. In the current study, we combined SEC with UF to isolate EVs. EVs isolated from the forebrain of the mouse were either collected acutely or after cells from the region were dissociated and grown in culture for 15 days. Acutely isolated EVs from the forebrain (hereinafter referred to as *in vivo* EVs) showed an approximate normal distribution between 50–250 nm in diameter with a major peak of abundant EVs of around 114 nm (Fig 1A). To compare *in vivo* EVs with the forebrain EVs released under cultured conditions, we prepared primary cell cultures from the forebrain of 16-day-old mouse embryos. Cultures were grown for 15 DIV before beginning EV collection for 24 hr. The EVs (hereinafter referred to as *in vitro* EVs) isolated from the culture media were also characterized by NTA. Results showed a more heterogeneous population of EVs, primarily sized between 20–250 nm in diameter, with a major peak at 133 nm and three smaller peaks at 24, 57, and 193 nm (Fig 1B). Comparatively, *in vitro* EVs yielded, on average, larger EVs but these EVs were reduced ~50% in concentration compared to *in vivo* EV isolates (Fig 1A and 1B).

### Proteome characterization of *in vivo* and *in vitro* EVs

Next, we sought to determine how the proteome of the *in vivo* and *in vitro* compared. The *in vivo* and *in vitro* EVs samples were then subjected to proteomic analysis by mass spectrometry to determine protein composition. A total of 3,204 proteins were identified across all *in vivo* EV samples while a total of 3,583 proteins were identified across all *in vitro* EVs samples (n = 4/group). A protein was considered expressed in either group of EVs if at least 2 replicates indicated expression of the protein. GO analysis was carried out on the proteins to determine the EV composition as indicated by CC, BP, and MF. Note that only terms resulting in p < 0.001 are displayed in (Fig 1C–1E). For CC GO enrichment (Fig 1C), a total of 29 terms were significantly enriched (p < 0.05 for Benjamini-Hochberg test) in *in vivo* EVs. The most notably enriched terms (p < 1e^-30^) include: cytoplasm (45.3% of 3,204), synapse (6.8%), cytoplasmic vesicles (7.8%), endosome (7.1%), and Golgi apparatus (9.1%). Similarly, a total of 28 CC GO terms (Fig 1C) were significantly enriched (p < 0.05) for *in vitro* EVs. The most notably enriched terms (p < 1e^-30^) were, again, for cytoplasm (45.3% of 3,583), synapse (6.3%), endosome (7.0%), Golgi apparatus (8.8%), and cytoplasmic vesicles (7.3%). For BP GO enrichment, a total of 45 terms (Fig 1D) were significantly enriched (p < 0.05) in *in vivo* EVs. The most notably enriched terms (p < 1e^-15^) included: protein transport (8.2%), transport (17.1%), mRNA processing (4.8%), mRNA splicing (4.1%), endoplasmic reticulum—Golgi transport (1.7%), and protein biosynthesis (2.2%). Similarly, again, a total of 49 BP GO terms (Fig 1D) were significantly enriched (p < 0.05) for *in vitro* EVs. The most notably enriched terms (p < 1e^-15^) were for protein transport (8.1%), transport (16.6%), mRNA processing (4.6%), mRNA splicing (3.8%), protein biosynthesis (2.0%), and endoplasmic reticulum—Golgi transport (1.7%). Finally, for MF GO enrichment, a total of 36 terms (Fig 1E) were significantly enriched (p < 0.05) in *in vivo* EVs. The most notably enriched terms (p < 1e^-10^) were for RNA-binding (7.9%), ribonucleoprotein (4.1%), actin-binding (3.1%), hydrolase (12.6%), chaperone (2.5%), ribosomal protein (2.5%), and ligase (1.9%). Again, a similar number (38 total) of MF GO terms (Fig 1E) were significantly enriched (p < 0.05) in *in vitro* EVs. The most notably enriched terms (p < 1e^-10^) were for RNA-binding (7.8%), ribonucleoprotein (3.9%), hydrolase (12.7%) actin-binding (2.9%), chaperone (2.3%), ligase (1.7%), and ribosomal protein (2.2%).

Further, 58 proteins were identified in our total sample proteome (containing both *in vivo* and *in vitro* samples) that corresponded with the term “extracellular exosome” (GO:0070062). This term contains a list of 154 genes, resulting in an approximate 38% overlap of our identified proteins with the GO term’s total list of proteins for “extracellular exosome”. When looking at the total proteome for *in vivo*, 57 proteins corresponded with the CC GO term “extracellular exosome” (p = 1e^-13^; 1.8%), while the total proteome of *in vitro* had 58 proteins corresponding to this term (p = 2.7e^-12^; 1.6%).

Additionally, KEGG pathway enrichment analysis was carried out on the identified proteins to determine molecular biological functions. A total of 128 pathways were found to be significantly enriched in the *in vivo* samples (p < 0.05, Benjamini-Hochberg) (Fig 1F). Note that only terms resulting in Benjamini-Hochberg p < 1e^-9^ are displayed in Fig 1F. The most notably enriched terms (p < 1e^-13^) included: endocytosis (3.6%), salmonella infection (3.2%), proteosome (1%), carbon metabolism (1.9%), and pathways of neurodegeneration in multiple diseases (4.9%). Similarly, a total of 129 terms were found to be significantly enriched in the *in vitro* samples (p < 0.05, Benjamini-Hochberg) (Fig 1F). The most notably enriched terms (p < 1e^-13^) included: endocytosis (3.5%), salmonella infection (3.1%), proteasome (1%), focal adhesion (2.5%), and carbon metabolism (1.8%).

Overall, the data indicates that the current combined method of SEC and UF isolates EVs released from *in vitro* primary dissociated brain cell culture medium that have a remarkably similar proteome as those released from *in vivo* EVs based on GO analysis and KEGG pathway analysis.

### *In vivo* and *in vitro* EVs proteome content comparison

**Fig 2:**
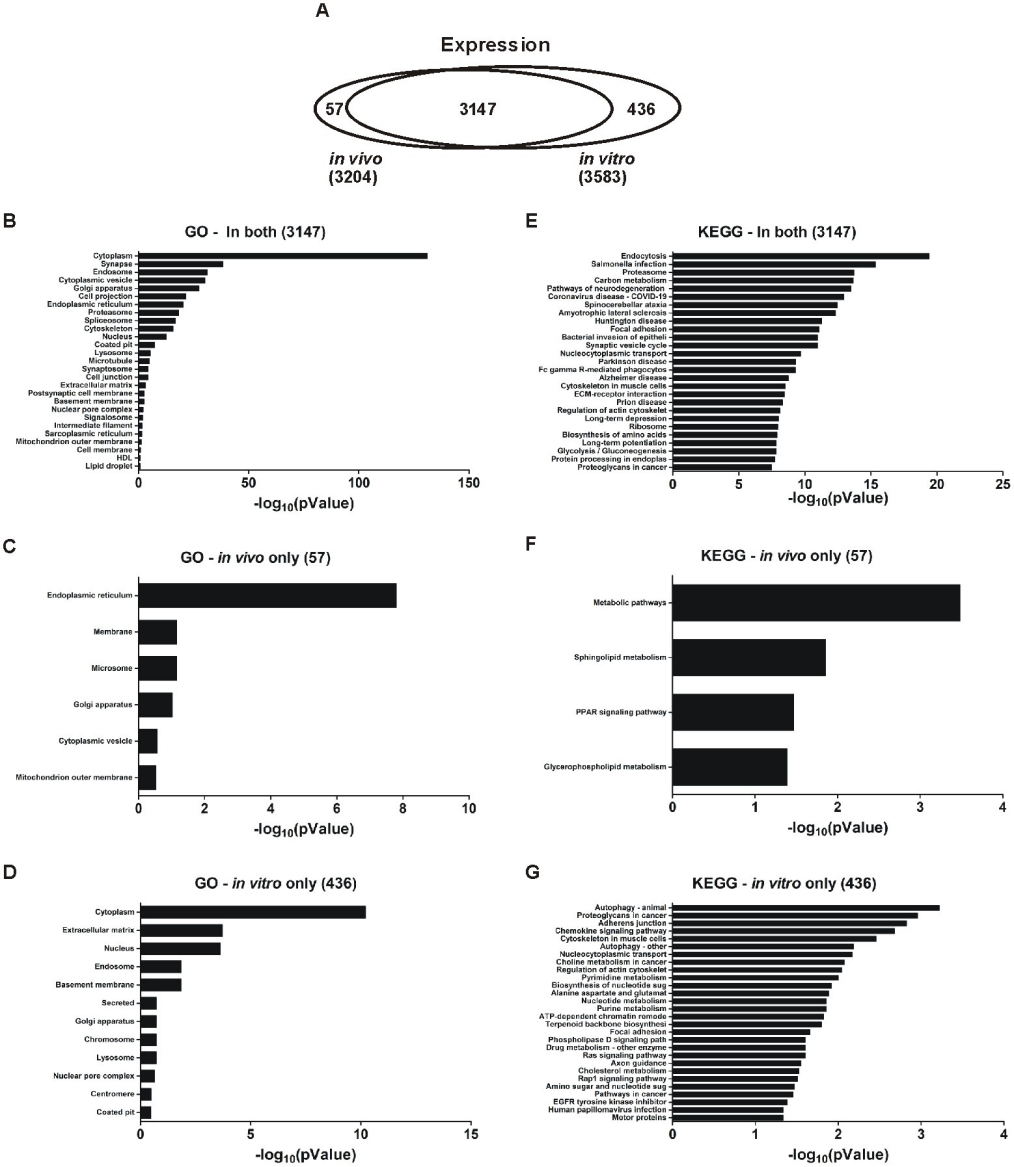
Comparison of protein composition between *in vivo* EVs and *in vitro* EVs. The proteomes for *in vivo* and *in vitro* EVs were overlaid to identify which proteins were expressed in both or were unique to one EV group. **(A)** A total of 3,204 and 3,583 proteins were identified *in vivo* and *in vitro* respectively. Of these proteins, 3,147 were expressed in both EV groups while only 57 proteins were exclusive to *in vivo* and 436 were exclusively identified *in vitro*. Data displayed is CC analysis of GO terms expressed **(B)** in both *in vivo* and *in vitro*, **(C)** only *in vivo*, and **(D)** only *in vitro*. (**E-G**) Data displayed is KEGG pathway enrichment analysis: (**E**) proteins found in both *in vivo* and *in vitro* EVs (p < 1e^-6^), (**F**) proteins found only *in vivo* EVs (p < 0.05), and (**G**) proteins found only *in vitro* EVs (p < 0.05). Data displayed for CC GO terms and KEGG pathways are the Benjamini-Hochberg p-value (**B-E**) and normal p-value (**F, G**) for term/pathway enrichment converted to -log10. All CC GO data shown is p < 0.05. KEGG pathway data shown is p < 0.05 (**F, G**) and p < 1e^-7^ (**E**). n = 4 replicates per EV group.

Next, we compared protein composition between *in vivo* EVs (3,204 proteins) and *in vitro* EVs (3,583 proteins). Overlap between *in vivo* and *in vitro* EVs are most likely to represent actual EV proteins. There were 3,147 proteins overlapped between *in vivo* EVs and *in vitro* EVs, representing 86.4% total overlap, with only 57 proteins found exclusively in the *in vivo* EVs (1.6%), and 436 proteins found exclusively in the *in vitro* EVs (12.0%; Fig 2A). CC GO enrichment analysis revealed that the 3,147 overlapping proteins were significantly enriched for 26 CC GO terms (*p*-value < 0.05). These top CC GO terms included the following (p < 0.001): cytoplasm (45.8% of all the overlapped 3,147 proteins), synapse (6.7%), endosome (7.1%), cytoplasmic vesicle (7.8%), Golgi apparatus (9.0%), cell projection (10.5%), endoplasmic reticulum (10.7%), protease (1.3%), spliceosome (2.3%), cytoskeleton (10.6%), nucleus (31.5%), coated pit (0.9%), lysosome (3.3%), microtubule (2.5%), synaptosome (0.8%), cell junction (3.2%), and ECM (2.1%; Fig 2B). This is consistent with the CC GO term enrichment for each EV proteome alone (Fig 1C). The 57 proteins found exclusively in the *in vivo* EVs were significantly enriched for 9 GO cellular component terms (p < 0.05). Of these CC GO terms (Fig 2C), endoplasmic reticulum was most robustly enriched (36.4% of 57 proteins; p < 0.001). The 436 proteins found exclusively in the *in vitro* EVs were significantly enriched for 12 CC GO terms (p < 0.05). The most enriched CC GO terms (Fig 2D) included: cytoplasm (41.2% of 436 proteins), ECM (4.4%), and nucleus (33.7%).

KEGG pathway analysis revealed that the 3,147 overlapping proteins were significantly enriched for 112 KEGG pathway terms (p < 0.05, Benjamini-Hochberg). The most notably enriched KEGG pathway terms (p < 1e^-13^) included: endocytosis (3.6%), salmonella infection (3.2%), proteosome (1.1%), carbon metabolism (1.9%), and pathways of neurodegeneration in multiple diseases (5%) (Fig 2E). The 57 proteins found exclusively in the *in vivo* EVs were significantly enriched for 4 KEGG pathway terms (p < 0.05) (Fig 2F). Of these KEGG pathway terms, metabolic pathways were the most robustly enriched (26.3%, p < 0.001), along with sphingolipid metabolism (5.3%), PPAR signaling pathway (5.3%), and glycerophospholipid metabolism (5.3%). The 436 proteins found exclusively in the *in vitro* EVs were significantly enriched for 28 KEGG pathway terms (Fig 2G; p < 0.05). The most enriched terms included: animal autophagy (2.9%), proteoglycans in cancer (3.1%), adherens junction (2%), chemokine signaling pathway (2.9%), and cytoskeleton in muscle cells (3.1%).

As the vast majority of proteins between the *in vivo* and *in vitro* EV groups were so similar, it is conceivable that all of these common proteins are associated with EVs and/or are representative of common contaminants associated with EVs. The limited number of proteins exclusively identified in *in vivo* EVs makes it difficult to determine what this small population of proteins represents but may be indicative of contaminants unique or selectively increased in *in vivo* EV isolation. The proteins exclusively identified in the *in vitro* EVs could be associated with the EVs that carry nuclear content or from ECM turnover as cells were not damaged during EV collection.

### *In vivo* and *in vitro* EVs proteomes have different relative expression

**Fig 3:**
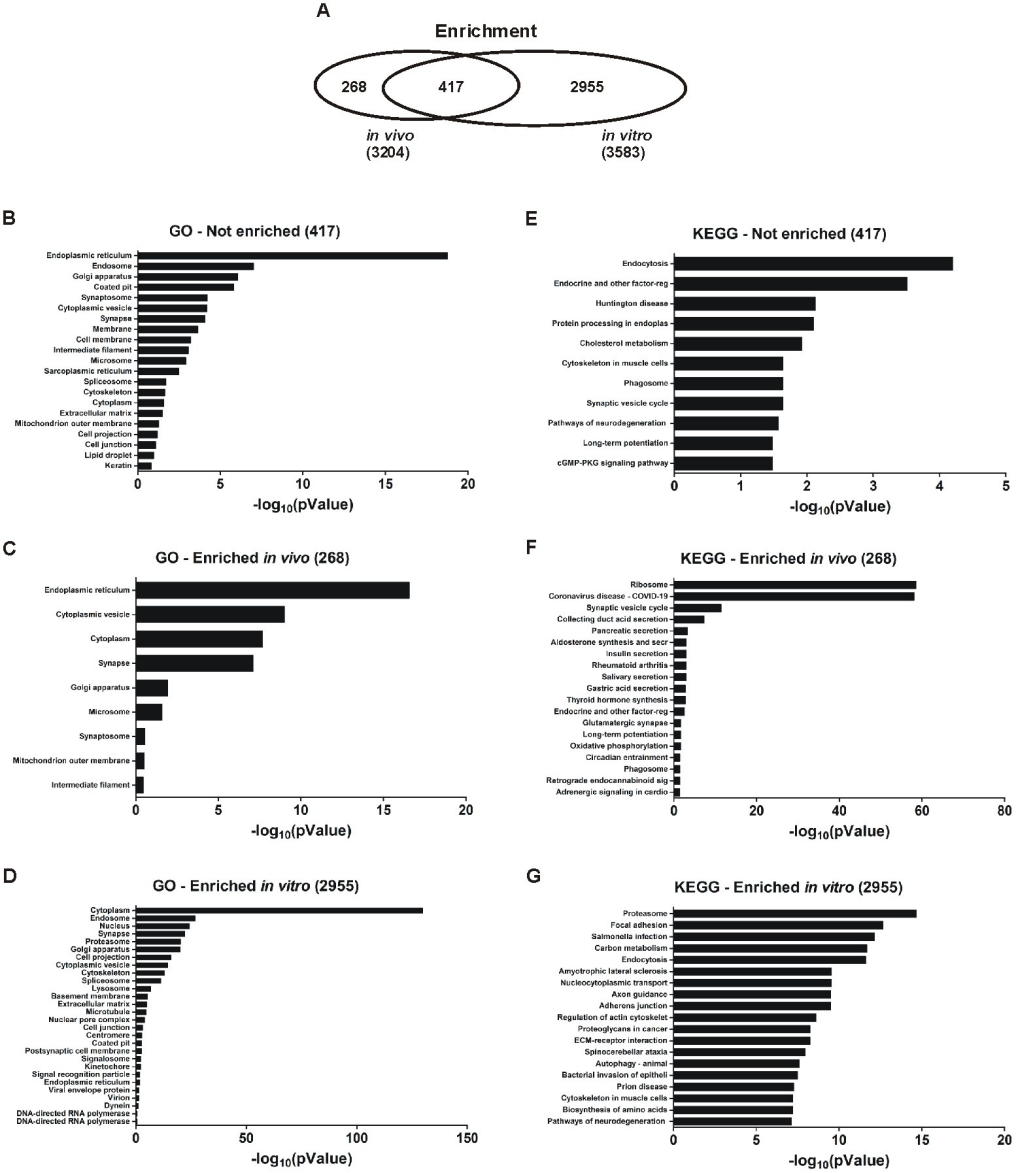
Proteome enrichment of *in vivo* and *in vitro* EVs. **(A-D)** As the overlap for both proteomes was largely conserved, the proteomes for *in vivo* and *in vitro* were compared for relative enrichment of proteins. Relative enrichment was determined by converting the LFQ mass spectrometry values to a Z-score for comparison between replicates and between groups. A two-tailed Student’s t-test was used to determine if a protein was enriched into either *in vivo* or *in vitro* EVs (p < 0.05) or was not enriched in either group (p ≥ 0.05). Proteins that were unique to either EV type were included for analysis. **(A)** Overall, only 417 proteins were similarly expressed in *in vivo* and *in vitro* EVs (p < 0.05). *In vitro* EVs were significantly enriched for most of the proteins analyzed (2,955, p < 0.05), while the *in vivo* EVs, again, only exhibited a few significantly enriched proteins (268, p < 0.05). Data displayed is CC analysis of GO terms that were **(B)** not enriched either *in vivo* or *in vitro* EVs, **(C)** enriched specifically in *in vivo* EVs, and **(D)** enriched specifically *in vitro* EVs. (**E-G**) Both enriched and not enriched proteins were further analyzed for KEGG pathway enrichment analysis. Proteins that were unique for either *in vivo* or *in vitro* EVs were included for analysis. Data displayed is KEGG pathway enrichment analysis of proteins that were (**E**) not enriched in either *in vivo* or *in vitro* EVs (p < 0.05), (**F**) specifically enriched in *in vivo* EVs (p < 0.05), and (**G**) specifically enriched in *in vitro* EVs (p < 1e^-7^). Data displayed for CC GO terms and KEGG pathways are the Benjamini-Hochberg p-value for term/pathway enrichment converted to -log_10_. All CC GO data shown is p < 0.05. KEGG pathway data shown is p < 0.05 (**E, F**) and p < 1e^-7^ (**G**). n = 4 replicates per EV group.

While the overall proteome content of both *in vivo* and *in vitro* EVs is mostly conserved, the relative abundance of individual proteins between the two sample types is quite disparate. To compare the enrichment of each protein between sample types, the individual LFQ values for each replicate were converted to a Z-score to describe the relative abundance of each protein in relation to the overall sample. The values of each replicate were then compared via a Student’s T-test (two-tailed) to determine if each protein is significantly enriched in either sample type (*in vivo* compared to *in vitro*; p < 0.05). Of the total 3,640 proteins identified when considering both *in vivo* and *in vitro* EVs, only 417 (11.5%) of the proteins were not significantly enriched (p ≥ 0.05, t-test of Z-scores abundancy values) in either sample type (Fig 3A). The composition of this category of proteins was assessed by CC GO analysis with 21 terms significantly enriched (p<0.05, Benjamini-Hochberg). The following terms represent the most robustly enriched CC GO terms (p < 0.001, Benjamini-Hochberg): endoplasmic reticulum (19.9% of 417 proteins), endosome (9.2%), Golgi apparatus (11.1%), coated pit (2.5%), synaptosome (2.3%), cytoplasmic vesicle (8.3%), synapse (6.7%), membrane (55.7%), cell membrane (26.1%), and intermediate filament (2.1%) (Fig 3B). Similar to the exclusive protein expression pattern (Fig 2A), only a small percentage of proteins were significantly enriched or exclusively expressed in the *in vivo* EVs (268 proteins; 7.4%; p < 0.05, t-test of abundancy Z-score abundancy value) while *in vitro* EVs (2,955 proteins) yielded a staggering 81.2% of enriched (2,519 proteins) or exclusive (436 proteins) protein expression (Fig 3A). For proteins enriched *in vivo*, there were 9 CC GO terms significantly enriched (p < 0.05, Benjamini-Hochberg) with the following terms representing the most robustly enriched (p < 0.001, Benjamini-Hochberg): endoplasmic reticulum (23.3% of 268 proteins), cytoplasmic vesicle (13.5%), cytoplasm (44.4%), and synapse (10.2%) (Fig 3C). For those proteins enriched *in vitro* there were 28 CC GO terms significantly enriched (p < 0.05, Benjamini-Hochberg) with the following terms representing the most robustly enriched (p < 0.001, Benjamini-Hochberg): cytoplasm (45.1% of 2,955 proteins), endosome (6.8%), nucleus (33.0%), synapse (5.7%), proteasome (1.3%), Golgi apparatus (8.3%), cell projection (9.7%), cytoplasmic vesicle (6.4%), cytoskeleton (10.1%), spliceosome (2.0%), lysosome (3.4%), basement membrane (0.7%), ECM (2.3%), microtubule (2.4%), nuclear pore complex (0.6%), and cell junction (2.9%) (Fig 3D).

In addition to the GO CC analysis, a KEGG pathway analysis was performed to assess these categories of proteins. There were 11 KEGG pathway terms (Fig 3E) that were not significantly enriched (p < 0.05, Benjamini-Hochberg) which included: endocytosis (5.5%), endocrine and other factor-regulated calcium reabsorption (2.5%), Huntington disease (4.8%), protein processing in endoplasmic reticulum (3.5%), cholesterol metabolism (1.8%), synaptic vesicle cycle (2.1%), phagosome (3.2%), cytoskeleton in muscle cells (3.7%), pathways of neurodegeneration in multiple diseases (5.8%), cGMP-PKG signaling pathway (3%), and long-term potentiation (1.8%). For proteins enriched *in vivo* EVs, there were 19 KEGG pathway terms that were significantly enriched (p < 0.05, Benjamini-Hochberg) with the following terms representing the most robustly enriched (p < 0.001, Benjamini-Hochberg): ribosome (25.5%), coronavirus disease (27.3%), synaptic vesicle cycle (6.5%), collecting duct acid secretion (3.6%), and pancreatic secretion (4.4%) (Fig 3F). For the proteins enriched *in vitro*, there were 97 KEGG pathway terms significantly enriched (p < 0.05, Benjamini-Hochberg) with the following terms representing the most robustly enriched (p < 1e^-10^, Benjamini-Hochberg): proteasome (1.2%), focal adhesion (2.8%), salmonella infection (3.2%), carbon metabolism (1.9%), and endocytosis (3.3%) (Fig 3G).

### Comparison of *in vivo* and *in vitro* EV proteins using MISEV2023 EV content categories and Vesiclepedia top 100 list

**Fig 4:**
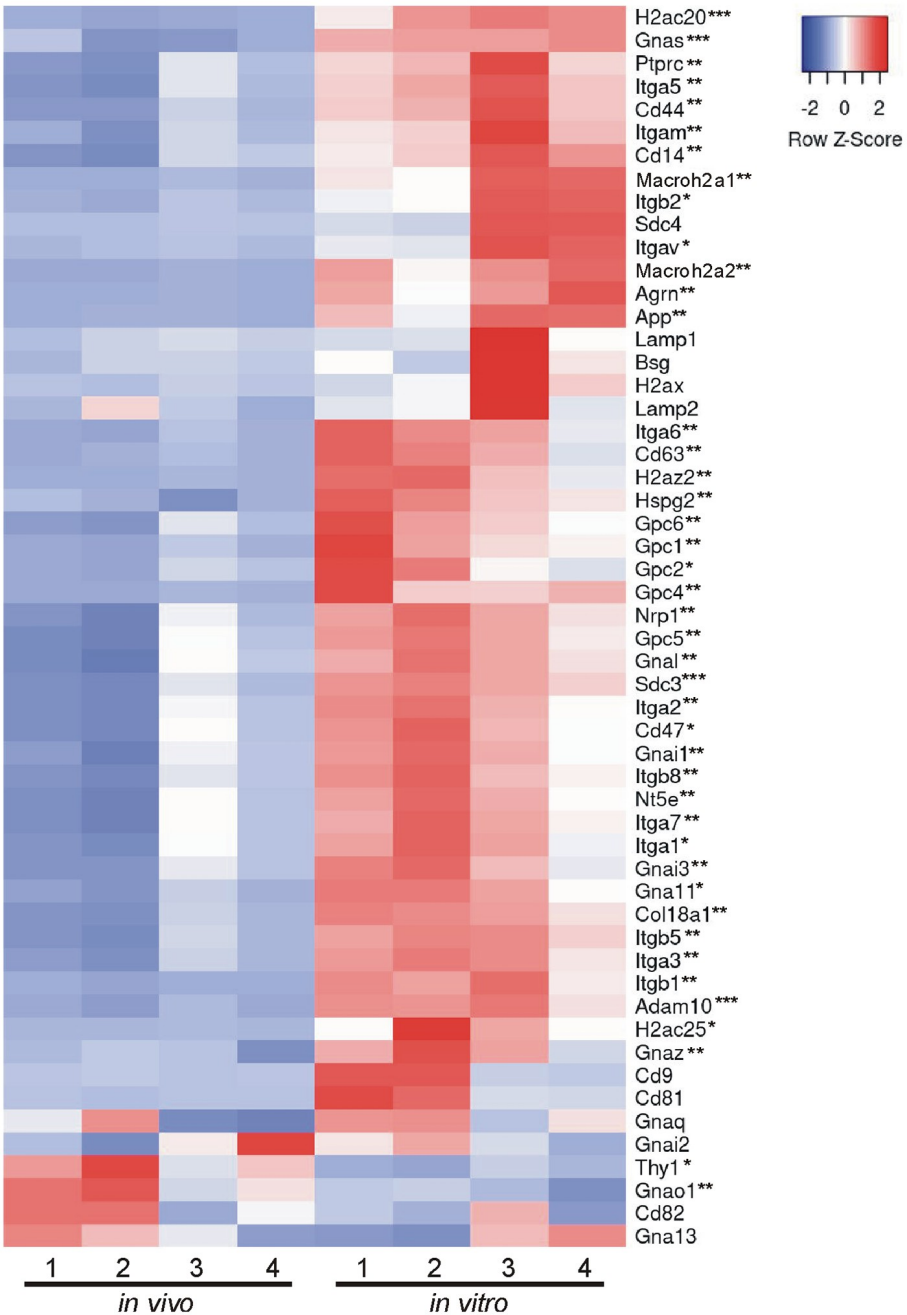
Enrichment of *in vivo* and *in vitro* EV proteins using MISEV2023 EV content category 1. Proteins identified in either or both EV groups were organized based on the MISEV2023 EV content categories for analysis. Relative enrichment for each protein in each category was determined by converting the LFQ mass spectrometry values to a Z-score for comparison between replicates and between groups. The results of the enrichment analysis are displayed as a heatmap. EV category 1 proteins are enriched *in vitro*. Heatmaps display the row Z-score values with red representing relative enrichment, blue indicating relative depletion, and white indicating no enrichment or depletion for either *in vivo* or *in vitro* EVs. A two-tailed Student’s t-test was used to determine if a protein was significantly enriched in either *in vivo* or *in vitro* EVs (p < 0.05) or was not enriched in either group (p ≥ 0.05). *p < 0.05, **p < 0.01, ***p < 0.001. n = 4 replicates per EV group.

**Fig 5:**
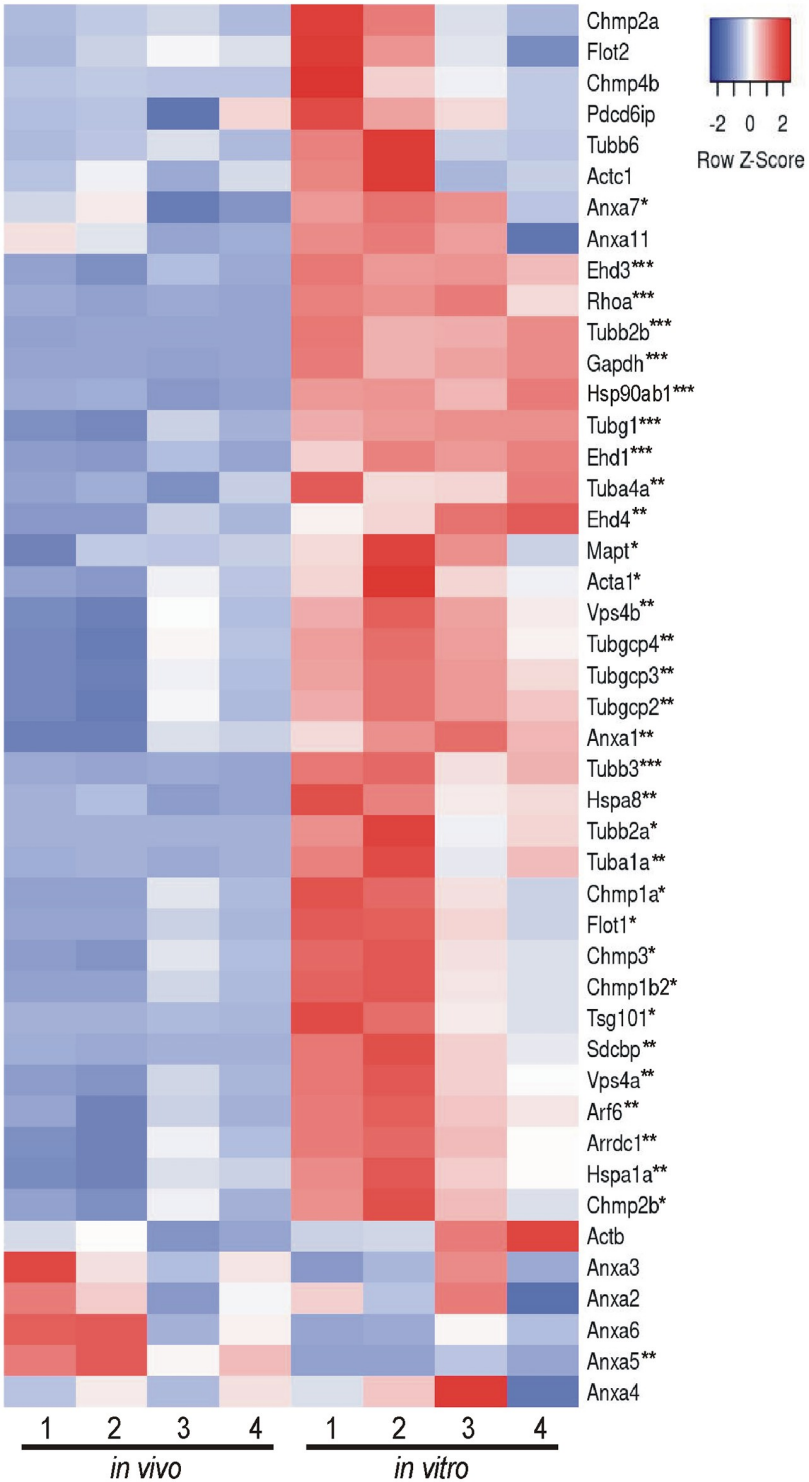
Enrichment of *in vivo* and *in vitro* EV proteins using MISEV2023 EV content category 2. Proteins identified were analyzed as described in the Fig 4 figure legend with EV category 2 proteins being enriched *in vitro*.

**Fig 6:**
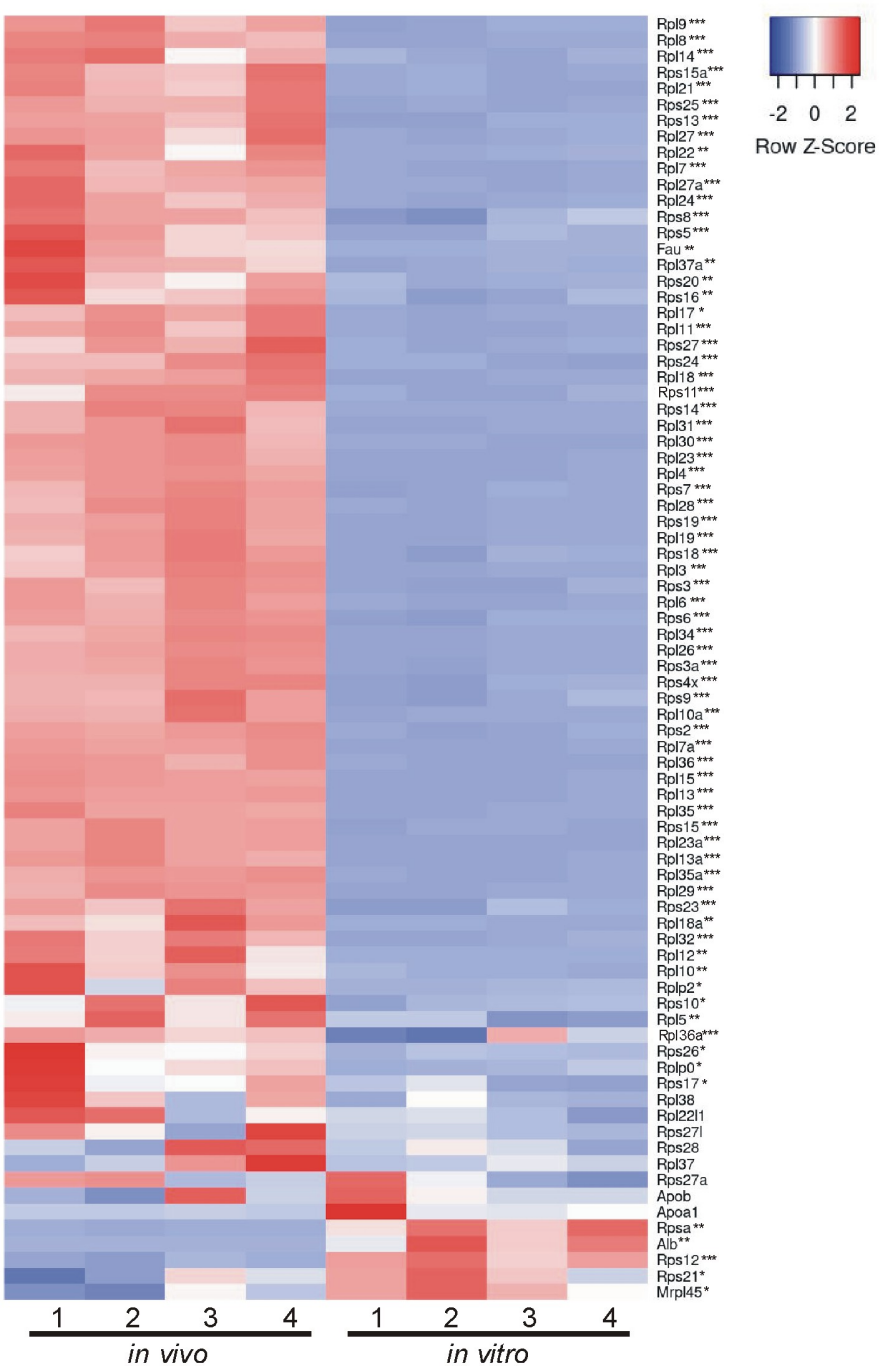
Enrichment of *in vivo* and *in vitro* EV proteins using MISEV2023 EV content category 3. Proteins identified were analyzed as described in the Fig 4 figure legend with EV category 3 proteins, considered to be likely co-elution contaminants, primarily enriched *in vivo*.

**Fig 7:**
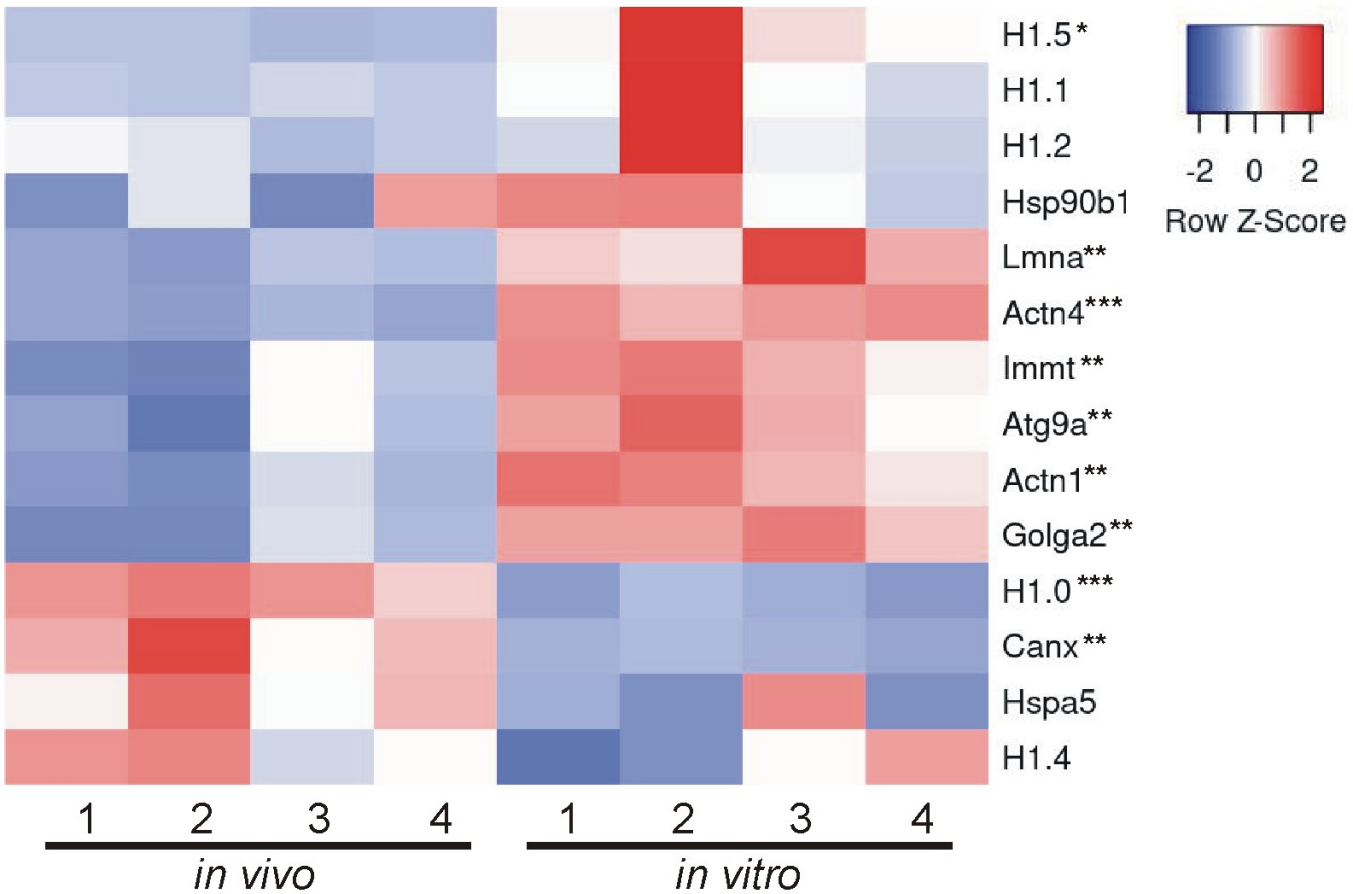
Enrichment of *in vivo* and *in vitro* EV proteins using MISEV2023 EV content category 4. Proteins identified were analyzed as described in the Fig 4 figure legend with EV category 4 proteins that may suggest EV subtypes were mostly enriched *in vitro*.

**Fig 8:**
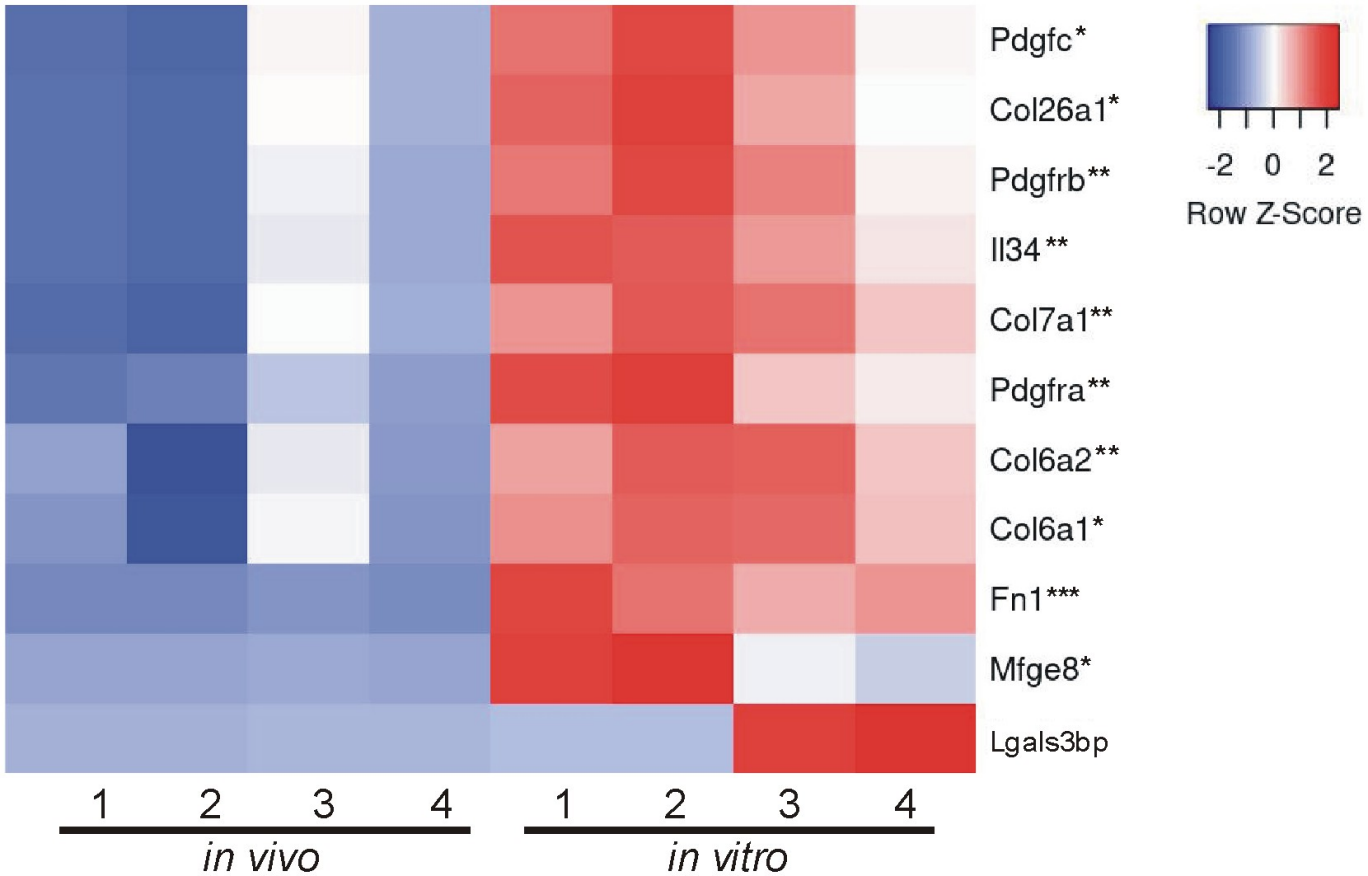
Enrichment of *in vivo* and *in vitro* EV proteins using MISEV2023 EV content category 5. Proteins identified were analyzed as described in the Fig 4 figure legend with EV category 5 proteins that may be used to assess possible EV function were mostly enriched *in vitro*.

Beyond looking exclusively at GO analysis and KEGG analysis to determine the composition and relative enrichment of protein categories in the EV proteomes, we further sought to compare each proteome with known EV and common EV contaminating proteins. To accomplish this, we utilized two categorical lists: (A) MISEV2023 established by the Executive Committee of the International Society for Extracellular Vesicles [28] and (B) the Vesiclepedia Top 100 proteins identified in EV studies [29]. First we created categorical lists of all proteins indicated in the MISEV2023 recommendations (including family homologues as indicated) for the following 5 categories: category 1, transmembrane or GPI-anchored proteins associated with plasma membrane and/or endosomes; category 2, cytosolic proteins recovered in EVs; category 3, major components of non-EV co-isolated structures; category 4, transmembrane, lipid bound and soluble proteins associated to intracellular compartments other than plasma membrane and/or endosomes; and category 5, secreted proteins recovered with EVs [28]. We then used the proteins from the 5 categorical lists and identified which proteins were expressed in our EV samples. Of those proteins identified, we next compared the relative abundance of each protein identified between *in vivo* and *in vitro* EVs. Results are presented in a heatmap for each category (Figs 4–8), which demonstrates the relative enrichment of each protein in the *in vivo* and *in vitro* EVs represented by the Z-score (converted from LFQ value as described above). A high Z-score (relative enrichment) is represented in red color indicating higher protein abundance versus the mean of all proteins in that individual sample, and a low Z-score (relative depletion) is represented by blue color indicating lower protein abundance versus the mean of all proteins in that independent sample. Further, the statistical p-values for average sample type enrichment of each protein, based on Student’s T-test of Z-score values, are indicated at the end of the protein name. Note the same strategy was used for analysis of the Vesiclepedia Top 100 proteins.

Fig 4 displays category 1 transmembrane and membrane bound proteins that are expected to be enriched in EVs. There were 52 proteins found to belong to category 1 expressed in the current EV data set. Overall, the majority of these proteins were significantly more abundant in the *in vitro* EVs as compared to the *in vivo* EVs (Fig 4). Notably, this included the tetraspanins Cd63 and Cd47; integrin proteins Itga1/3/5-7/m/v and Itgb1/2/5/8; and heterotrimeric G proteins Gnas, Gnai1/3, Gna11/l/z. Interestingly, the common, canonical EV proteins Cd9, Cd81, Cd82 and Bsg did not show significant enrichment in either EV type indicating equivalent relative expression.

Fig 5 displays category 2 cytosolic proteins that are expected to be enriched in EVs. There were 42 proteins found to belong to category 2. Consistent with category 1 proteins, category 2 proteins continued to display significant enrichment in the *in vitro* EVs relative to the *in vivo* EVs. Importantly, these proteins included the canonical EV proteins: endosomal sorting proteins Tsg101, Vps4a, Arrdc1, and Chmpa1a/1b2/2b/3; Flot1; Anxa1/7; heat shock proteins, Hspa8 and Hsp90ab1; and structural proteins Tubb1a/2a/3 among others.

Fig 6 displays category 3 co-eluted proteins that are expected to be contaminants or underrepresented in EVs, *e*.*g*., lipoproteins and ribosomal proteins. There were 79 category 3 likely contaminant proteins identified which were almost exclusively enriched in *in vivo* EV samples. Specifically, many components of the 40S small (Rps* proteins) and the 60S large (Rpl* proteins) subunits of the ribosome complex were robustly enriched *in vivo*. In regard to ribosomal proteins, only Rps12 and Rps21 were preferentially enriched *in vitro*. Of note, the plasma protein Alb was significantly enriched *in vitro* suggesting some contamination remaining from the serum containing media used for maintaining the cells *in vitro* before switching to serum free media for EV collection.

Fig 7 displays category 4 proteins that may be used to show EV subtype or cellular origin. A total of 13 proteins from this category were identified with 8 showing significant enrichment. However, the enrichment pattern was mixed between *in vivo* and *in vitro* (although *in vitro* still exhibited more proteins significantly enriched than *in vivo*). Both *in vivo* and *in vitro* EVs were enriched for nuclear proteins which suggests that these EVs may have a nuclear origin (H1.0 and H1.5/Lmna/Actn1/Actn4 respectively) or are carrying nuclear originating material. However, the endoplasmic reticulum protein calnexin (Canx) showed significant enrichment *in vivo* while the Golgi apparatus protein golgin A2 (GM130; Golga2) showed significant enrichment in *in vitro* suggesting additional origins for both EV types. Overall, the exact intracellular origin of the EVs from within the cell was indeterminant and not the focus of the current study as all EVs were collected from cells originating from the cells in the brain.

Fig 8 displays category 5 proteins which may represent functional signaling components of EVs. While only 9 proteins from this category were identified, 8 of these proteins were selectively enriched in the *in vitro* EVs. The proteins include those associated with growth factors (Pdgfrb and Mfge8), collagen production or the ECM (Col6a1, Col6a2, Col26a1, and Fn1), and pro-inflammatory cytokine release (Il34). Overall, the potential specific function of the EVs isolated here could not be determined and was not the focus of the current study.

**Fig 9:**
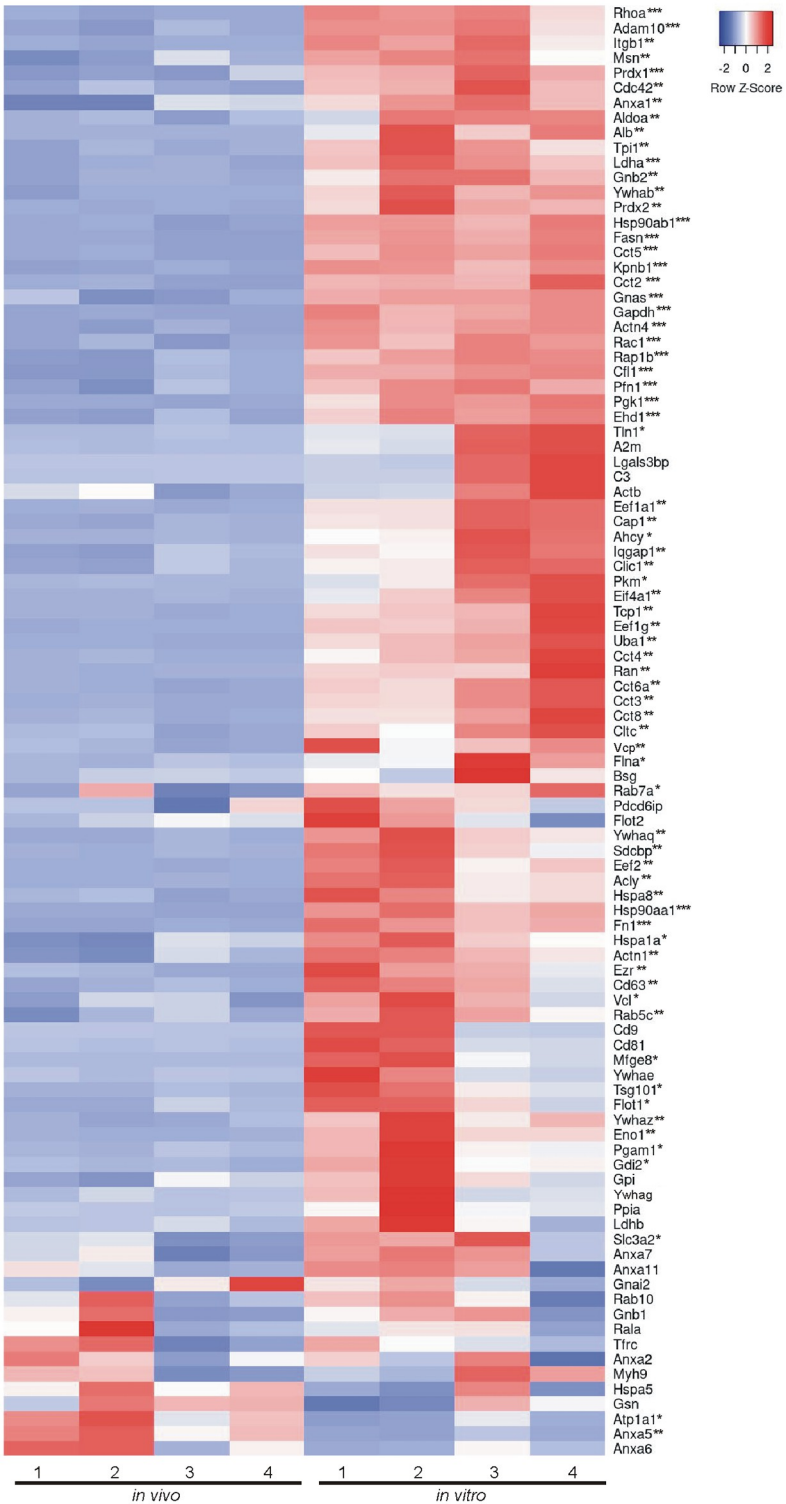
Enrichment of *in vivo* and *in vitro* EV proteins using Vesiclepedia top 100 list. The top 100 most often identified EV proteins were extracted from the Vesiclepedia database [29] and compared for relative enrichment between *in vivo* and *in vitro* EVs. Of these proteins, 97/100 were found to be expressed in the overlapping experimental EV proteome and were used for subsequent analysis. Overall, the *in vitro* EVs showed the most significant enrichment of the proteins assessed with 41/97 showing significant enrichment while only 2/97 were significantly enriched *in vivo*. However, it is important to note that 54/97 showed no significant enrichment and that all 97 proteins listed were identified both *in vivo* and *in vitro*. This data is represented in the format of a heatmap where red represents relative enrichment, blue indicates relative depletion, and white indicates no enrichment or depletion for either *in vivo* or *in vitro* EVs (based on row Z-scores). A two-tailed Student’s t-test was used to determine if a protein was significantly enriched in either *in vivo* or *in vitro* EVs (p < 0.05) or was not enriched in either group (p ≥ 0.05). *p < 0.05, **p < 0.01, ***p < 0.001. n = 4 replicates per EV group.

In addition to the MISEV2023 method of categorizing EV proteins, we further compared the proteins identified in the present study (both *in vivo* and *in vitro* EVs) with the Vesiclepedia top 100 listed proteins [29], which are proteins most often reported in EV studies. Of the Top 100 listed proteins, we were able to identify 97 in our dataset (only Hist1h4a, Hla-a, and Tubb4b were not identified). In accordance with the results found for MISEV2023 category 1 and 2 proteins, most of the Top 100 commonly found EV proteins were either preferentially or significantly enriched in the *in vitro* EVs (Fig 9). Of all 100 proteins, only Atp1a1 and Anxa5 were significantly enriched in the *in vivo* EVs. This result further indicates that *in vitro* EVs are selectively enriched for EV proteins or are selectively depleted of non-EV contaminants relative to *in vivo* EVs isolated in a similar manner from a similar source.

Characterizing the protein composition of EVs is important to study the functional impact of EVs [28, 37, 39, 40]. Isolating EVs from cell culture medium is a common approach to identify EV proteins considering many of the EV proteins were originally identified in culture systems. However, the extent to which culture conditions accurately reflect the physiological conditions of tissues with regard to EV protein composition remains unclear. Here, we present an in-depth analysis of the protein composition of brain EVs isolated from *in vivo* mouse forebrains and *in vitro* primary cells dissociated from the mouse forebrain to address this knowledge gap. We first characterized the size of isolated vesicles and found that the most prevalent size of vesicles from each source were similar, but not identical (131.5 vs 163.4, *in vivo* to *in vitro*). Further, most of the proteins (~86%), including 97 of the top 100 most commonly identified EV proteins, were expressed in both EV groups. However, a notable subset of proteins was exclusively identified *in vitro* and were composed mainly of cytoplasmic, ECM, and nuclear proteins. Contrastingly, very few proteins were found to be exclusively identified *in vivo*; however, those that were identified were enriched with endoplasmic reticulum proteins suggesting a source of contamination in *in vivo* EVs. Importantly, while the total proteome for both pools of EV were remarkably similar in terms of proteins expressed, the relative abundance of each protein between *in vivo* and *in vitro* EVs was vastly different. The *in vitro* EVs indicated a clear enrichment of canonical EV proteins. When the relative expression of all proteins was categorized into MISEV2023 category 1 and 2 (Figs 4 and 5), as well as the Vesiclepedia Top 100 proteins (Fig 9), *in vitro* EVs consistently showed enrichment for these proteins as compared to *in vivo* EVs. Further, *in vitro* EVs showed very robust depletion of MISEV2023 category 3 proteins, which represent co-eluting, non-EV contaminants, specifically indicating contamination from ribosomes in the *in vivo* EVs (Fig 6). Overall, the similarity of total proteome of both *in vivo* and *in vitro* EVs suggest that both EVs may be similar in function, but that they are not identical, especially as there is a clear difference in relative expression of canonical EV proteins, as well as probable sources of contamination between the 2 types of brain EVs. This should be considered when interpreting results between EVs collected from acute dissociation of intact tissues versus cell culture systems.

To isolate EVs from the brain *in vivo*, it is necessary to disrupt the tissue to facilitate release of EVs from the ECM that can insnare EVs released from the cells of origin. Our approach, commonly used in the field, involved gentle mechanical disruption to break the tissue into small pieces, followed by collagenase treatment. Collagenase is an enzyme commonly used to facilitate the dissociation of cells and EVs in larger tissues by cleaving collagen peptide bonds breaking down the ECM [2]. However, this gentle mechanical disruption can still cause cellular damage resulting in contamination of plasma membrane and intracellular organelles, which may explain why a large number of ribosomal contaminating proteins were enriched *in vivo* and not *in vitro*.

We used SEC combined with UF to enrich EVs isolated from brain tissue and cell culture medium. SEC is a gentle isolation method that reduces the risk of damage to the isolated EVs. It is important to note that each EV isolation method can affect the composition of the isolated EVs and co-isolated entities, which may vary depending on the sample type. Veerman *et al*., conducted a comparison of several EV isolation methods in cell culture and plasma samples [37]. In cell culture, they found that SEC yielded the highest number of EV associated MISEV2023 category 1 transmembrane/GPI proteins, but also co-isolated a greater amount of MISEV2023 category 3 non-EV proteins, especially lipoproteins. In human plasma, SEC isolated the fewest EV proteins but had a high presence of tetraspanins. These findings indicate that the efficiency of EV isolation can vary depending on the sample type as well as the isolation method, which may explain the differences in EV protein enrichment observed *in vitro* versus *in vivo* EVs in the current study.

While SEC is an increasingly common and viable method to rapidly isolate EVs from various sources, it is not, however, able to efficiently isolate EVs from other similarly sized particles, which can lead to contamination and interfere with downstream analyses [11]. For instance, EVs enriched using SEC may still contain significant amounts of other lipoprotein particles, especially when plasma or serum is the source, such as chylomicrons (100–600 nm) and very low-density lipoproteins (30–80 nm), as well as microvesicles (100–1000 nm) from other cellular compartments that are in the non-exclusion size range of the respective column [41–43]. In our study, we identified an enrichment of proteins associated with the nucleus and ER in the *in vitro* and *in vivo* EV samples suggesting that vesicles from these cellular compartments were co-isolated with the EVs. It is yet to be definitively determined whether these represent bona fide EV proteins or are sources of non-EV contaminants. Further, in the current study, UF was used not only to concentrate the large volumes produced by SEC, but to also deplete contaminating free proteins and very small vesicles (<10 nm). However, this method is ineffective at reducing contamination from similarly sized particles (>30 nm) that will co-elute using SEC. In the future, attempts should be made to further purify EVs from potential contaminating sources by adding an additional step to the SEC with UF (and likely any other) method used. For example, adding an immunoprecipitation step using antibodies that recognize the extracellular-facing EV-associated tetraspanin proteins CD9, CD63, and CD81 following SEC may substantially enrich EVs by further depleting contaminating proteins and similar sized particles. Any single-step (and possibly two-step) method is unlikely to adequately purify EVs for analysis of low expression proteins that may be masked by abundant contaminants.

Intracellular proteins with membrane binding ability are often co-isolates of EVs. An example of this is the Rab GTPase family which is a small GTP-binding protein that plays an important role in intracellular vesicle and EV trafficking [44]. Rab GTPases are involved in several steps of EV biogenesis and release, including trafficking of EVs through the endosomal system, release of EVs from the plasma membrane, and fusion of EVs with target cells [45]. Specific Rab GTPases are involved in EV biogenesis and trafficking, and the expression and activity levels of these Rab GTPases can alter the quantity and quality of EVs released. For example, Rab27a and Rab27b have been shown to be involved in the secretion of EVs from different cell types [46]. Interestingly, we only identified Rab27b in the current study of brain derived EVs. Rab11 has also been implicated in the trafficking of EVs from the recycling endosome to the plasma membrane for release [47]. Additionally, Rab35 has been shown to be involved in the biogenesis of EVs derived from multivesicular bodies (MVBs) [48]. In the current study, we detected a total of 29 Rab GTPase proteins common to both EV types. Most of these proteins were selectively enriched in *in vitro* EVs including: Rab- 3a, 3c,4a, 4b, 5b, 5c, 6a, 6b, 7a, 8a, 8b, 9a, 9b, 21, 22a, 23, 24, and 27b. Further, 7 Rab GTPase proteins were only identified in *in vitro* including: Rab- ac1, l6, 3 12, 32, 33b, and 40c. Not all Rab GTPases were enriched in either group, including: Rab- 1a, 1b, 2a, 5a, 10, 11b, 14, 18, and 35. Interestingly, no Rab GTPases were selectively enriched *in vivo*. Previous studies demonstrated that Rab- 4a, 5b, 5c, 7a, 22a, and 27b (expressed *in vivo* and *in vitro*, but enriched *in vitro* EVs) were enriched in small EVs (defined as vesicles enriched at 118,000 × g) compared to large EVs (defined as vesicles enriched at 16,500 × g) [48]. Both Rab1b and 18 (not enriched *in vivo* or *in vitro* in the current study) were found to be enriched in large EVs compared to small EVs. This suggests that EVs isolated from both sources likely have some large EV contamination but that the *in vitro* EVs are more representative of small EVs even though *in vitro* EVs were slightly larger in diameter on average.

## Conclusions

Our study provides insight into the protein composition of EVs isolated from similar *in vivo* and *in vitro* neuronal systems via SEC and UF. We found that there was substantial overlap between the proteomes of EVs from both sources (~86%). This suggests these overlapped proteins are likely core proteins associated with EVs originating from the brain with MISEV2023 and Vesiclepedia Top 100 supporting this conclusion. Conversely, it is also possible that some of these overlapped proteins may represent a common contamination source that is not amenable to SEC or UF purification. Together, it appears that both systems are amenable to EV isolation by SEC and therefore may be used to assess basic EV biology and translational biomarkers as each will generate similar core EV populations. However, it is important to indicate that *in vivo* and *in vitro* systems also differ significantly as the majority of the proteome exhibits differences in relative enrichment of individual proteins. The current study indicates that *in vitro* yields more canonical EV protein enrichment. Alternatively, the data could be interpreted as canonical EV proteins being more enriched *in vitro* due to less non-EV contamination. This is supported by increased contamination of ribosomal proteins noted in *in vivo* EVs. Overall, the results herein indicate that *in vitro* isolated EVs may be preferred to better understand the biochemical makeup of EVs from a neuronal tissue source as they seem to be more pure with less contamination. However, the current study also suggests that *in vivo* EVs can be readily utilized in lieu of *in vitro* as the 2 sample types have similar proteome composition, but with the consideration that lower abundant proteins may be difficult to detect due to increased contamination. Of note, MF GO analysis of overlapped proteins indicated many RNA binding proteins in the EVs. This indicates that additional study should be performed to assess the RNA and DNA content of each EV group to further assess the likeness of *in vivo* and *in vitro* EVs.

## Supporting information

S1 Fig EV isolation process. The figure gives a condensed pictorial outline of the EV collection and isolation methods from both *in vivo* and *in vitro* sources outlined in the Materials and Methods section of this manuscript.
(PDF)

S2 Fig Western blot analysis of *in vivo* EV isolation via SEC column and ultracentrifugation. These results showcase Western Blot analysis of *in vivo* EV isolation via SEC column and ultracentrifugation. Normalized amounts of protein were resolved by SDS—PAGE (8% or 10% polyacrylamide gel) and transferred onto nitrocellulose membranes. Membranes were probed against antibodies of interest including: TSG101 (ThermoFisher, MA1-23296), CD81 (ThermoFisher, MA5-32333), and Alix (Cell signaling, 2171S). Subsequently, goat anti-rabbit IgG or anti-mouse IgG secondary antibodies (Bio-Rad; 1:5,000) conjugated to horseradish-peroxidase were incubated with the blot. For visualization of immunoreactive bands, either WesternBright or WesternBright Sirius (Advansta) enhanced chemiluminescence substrates were applied according to the manufacturer’s directions. Digital images of immunoblots were captured using the ChemiDoc Imaging System (Bio-Rad) and band intensities were analyzed using Image Lab (Bio-Rad). Samples are labeled by “sample type-number(addendum).” P1 indicates the pellet of the second centrifugation (2,000 x g for 10 min) post-homogenization and dissociation. Whereas, TCL in this case refers to the supernatant of the first centrifugation (300 x g for 5 min) post-homogenization and dissociation, as this low speed spin was used exclusively to remove excess tissue debris and undissociated tissue remnants. Samples labeled EV are in reference to samples that underwent the entire EV isolation process through ultracentrifugation and SEC column isolation. The addendum “(a)” indicates the EV samples that were processed by a trainee and did not contain enough signal to showcase CD81, potentially due to user error. Western Blot analysis is not shown for *in vitro* EV isolation due to weak signaling from the cell culture samples, but EV markers were confirmed via mass spectroscopy and comparison with the MISEV2023 recommendations categories and Vessiclepedia Top100 list of most often identified EV proteins as seen in Figs 4 and 5, respectively, in the main body of this manuscript.
(PDF)

## References

[1] Akers JC, Gonda D, Kim R, Carter BS, Chen CC. Biogenesis of extracellular vesicles (EV): exosomes, microvesicles, retrovirus-like vesicles, and apoptotic bodies. J Neurooncol. 2013;113: 1–11. doi: 10.1007/s11060-013-1084-8 23456661 PMC5533094

[2] Brenna S, Krisp C, Altmeppen HC, Magnus T, Puig B. Brain-Derived Extracellular Vesicles in Health and Disease: A Methodological Perspective. IJMS. 2021;22: 1365. doi: 10.3390/ijms22031365 33573018 PMC7866382

[3] Kumar A, Nader MA, Deep G. Emergence of Extracellular Vesicles as “Liquid Biopsy” for Neurological Disorders: Boom or Bust. Baker A, editor. Pharmacol Rev. 2024;76: 199–227. doi: 10.1124/pharmrev.122.000788 38351075 PMC10877757

[4] Santiago-Dieppa DR, Steinberg J, Gonda D, Cheung VJ, Carter BS, Chen CC. Extracellular vesicles as a platform for ‘liquid biopsy’ in glioblastoma patients. Expert Review of Molecular Diagnostics. 2014;14: 819–825. doi: 10.1586/14737159.2014.943193 25136839 PMC4436244

[5] Badhwar A, Haqqani AS. Biomarker potential of brain‐secreted extracellular vesicles in blood in Alzheimer’s disease. Alzheimer’s & Dementia: Diagnosis, Assessment & Disease Monitoring. 2020;12. doi: 10.1002/dad2.12001 32211497 PMC7085285

[6] Qian F, Huang Z, Zhong H, Lei Q, Ai Y, Xie Z, et al. Analysis and Biomedical Applications of Functional Cargo in Extracellular Vesicles. ACS Nano. 2022;16: 19980–20001. doi: 10.1021/acsnano.2c11298 36475625

[7] Hagey DW, El Andaloussi S. The promise and challenges of extracellular vesicles in the diagnosis of neurodegenerative diseases. Handbook of Clinical Neurology. Elsevier; 2023. pp. 227–241. 10.1016/B978-0-323-85555-6.00014-X 36803813

[8] Xu X, Iqbal Z, Xu L, Wen C, Duan L, Xia J, et al. Brain‐derived extracellular vesicles: Potential diagnostic biomarkers for central nervous system diseases. Psychiatry Clin Neurosci. 2024;78: 83–96. doi: 10.1111/pcn.13610 37877617

[9] De Sousa KP, Rossi I, Abdullahi M, Ramirez MI, Stratton D, Inal JM. Isolation and characterization of extracellular vesicles and future directions in diagnosis and therapy. WIREs Nanomed Nanobiotechnol. 2023;15: e1835. doi: 10.1002/wnan.1835 35898167 PMC10078256

[10] Askeland A, Borup A, Østergaard O, Olsen JV, Lund SM, Christiansen G, et al. Mass-Spectrometry Based Proteome Comparison of Extracellular Vesicle Isolation Methods: Comparison of ME-kit, Size-Exclusion Chromatography, and High-Speed Centrifugation. Biomedicines. 2020;8: 246. doi: 10.3390/biomedicines8080246 32722497 PMC7459681

[11] Brennan K, Martin K, FitzGerald SP, O’Sullivan J, Wu Y, Blanco A, et al. A comparison of methods for the isolation and separation of extracellular vesicles from protein and lipid particles in human serum. Sci Rep. 2020;10: 1039. doi: 10.1038/s41598-020-57497-7 31974468 PMC6978318

[12] Stranska R, Gysbrechts L, Wouters J, Vermeersch P, Bloch K, Dierickx D, et al. Comparison of membrane affinity-based method with size-exclusion chromatography for isolation of exosome-like vesicles from human plasma. J Transl Med. 2018;16: 1. doi: 10.1186/s12967-017-1374-6 29316942 PMC5761138

[13] Turner NP, Abeysinghe P, Kwan Cheung KA, Vaswani K, Logan J, Sadowski P, et al. A Comparison of Blood Plasma Small Extracellular Vesicle Enrichment Strategies for Proteomic Analysis. Proteomes. 2022;10: 19. doi: 10.3390/proteomes10020019 35736799 PMC9229025

[14] Ansari FJ, Tafti HA, Amanzadeh A, Rabbani S, Shokrgozar MA, Heidari R, et al. Comparison of the efficiency of ultrafiltration, precipitation, and ultracentrifugation methods for exosome isolation. Biochemistry and Biophysics Reports. 2024;38: 101668. doi: 10.1016/j.bbrep.2024.101668 38405663 PMC10885727

[15] Jimenez DE, Tahir M, Faheem M, Alves WBDS, Correa BDL, Andrade GRD, et al. Comparison of Four Purification Methods on Serum Extracellular Vesicle Recovery, Size Distribution, and Proteomics. Proteomes. 2023;11: 23. doi: 10.3390/proteomes11030023 37606419 PMC10443378

[16] Stam J, Bartel S, Bischoff R, Wolters JC. Isolation of extracellular vesicles with combined enrichment methods. Journal of Chromatography B. 2021;1169: 122604. doi: 10.1016/j.jchromb.2021.122604 33713953

[17] Prada I, Gabrielli M, Turola E, Iorio A, D’Arrigo G, Parolisi R, et al. Glia-to-neuron transfer of miRNAs via extracellular vesicles: a new mechanism underlying inflammation-induced synaptic alterations. Acta Neuropathol. 2018;135: 529–550. doi: 10.1007/s00401-017-1803-x 29302779 PMC5978931

[18] You Y, Muraoka S, Jedrychowski MP, Hu J, McQuade AK, Young‐Pearse T, et al. Human neural cell type‐specific extracellular vesicle proteome defines disease‐related molecules associated with activated astrocytes in Alzheimer’s disease brain. J of Extracellular Vesicle. 2022;11: e12183. doi: 10.1002/jev2.12183 35029059 PMC8758831

[19] Almansa D, Peinado H, García-Rodríguez R, Casadomé-Perales Á, Dotti CG, Guix FX. Extracellular Vesicles Derived from Young Neural Cultures Attenuate Astrocytic Reactivity In Vitro. IJMS. 2022;23: 1371. doi: 10.3390/ijms23031371 35163295 PMC8835866

[20] Rodríguez RG, Cadavieco MC, Guix F, Dotti C. YOUNG NEURAL EXTRACELLULAR VESICLES INCREASE SURVIVAL IN AGED NEURAL CELL CULTURES AND PROTECT AGAINST INFLAMMATORY STIMULI. IBRO Neuroscience Reports. 2023;15: S408–S409. doi: 10.1016/j.ibneur.2023.08.780

[21] Aulston B, Liu Q, Mante M, Florio J, Rissman RA, Yuan SH. Extracellular Vesicles Isolated from Familial Alzheimer’s Disease Neuronal Cultures Induce Aberrant Tau Phosphorylation in the Wild-Type Mouse Brain. JAD. 2019;72: 575–585. doi: 10.3233/JAD-190656 31594233 PMC8373022

[22] Peng H, Harvey BT, Richards CI, Nixon K. Neuron-Derived Extracellular Vesicles Modulate Microglia Activation and Function. Biology. 2021;10: 948. doi: 10.3390/biology10100948 34681047 PMC8533634

[23] Beretta C, Nikitidou E, Streubel-Gallasch L, Ingelsson M, Sehlin D, Erlandsson A. Extracellular vesicles from amyloid-β exposed cell cultures induce severe dysfunction in cortical neurons. Sci Rep. 2020;10: 19656. doi: 10.1038/s41598-020-72355-2 33184307 PMC7661699

[24] You Y, Borgmann K, Edara VV, Stacy S, Ghorpade A, Ikezu T. Activated human astrocyte‐derived extracellular vesicles modulate neuronal uptake, differentiation and firing. J of Extracellular Vesicle. 2020;9: 1706801. doi: 10.1080/20013078.2019.1706801 32002171 PMC6968484

[25] Chun C, Smith AST, Kim H, Kamenz DS, Lee JH, Lee JB, et al. Astrocyte-derived extracellular vesicles enhance the survival and electrophysiological function of human cortical neurons in vitro. Biomaterials. 2021;271: 120700. doi: 10.1016/j.biomaterials.2021.120700 33631652 PMC8044026

[26] Théry C, Amigorena S, Raposo G, Clayton A. Isolation and Characterization of Exosomes from Cell Culture Supernatants and Biological Fluids. CP Cell Biology. 2006;30. doi: 10.1002/0471143030.cb0322s30 18228490

[27] Crescitelli R, Lässer C, Lötvall J. Isolation and characterization of extracellular vesicle subpopulations from tissues. Nat Protoc. 2021;16: 1548–1580. doi: 10.1038/s41596-020-00466-1 33495626

[28] Welsh JA, Goberdhan DCI, O’Driscoll L, Buzas EI, Blenkiron C, Bussolati B, et al. Minimal information for studies of extracellular vesicles (MISEV2023): From basic to advanced approaches. J of Extracellular Vesicle. 2024;13: e12404. doi: 10.1002/jev2.12404 38326288 PMC10850029

[29] Vesiclepedia. In: Extracellular vesicle Markers [Internet]. [cited 29 Jan 2024]. http://microvesicles.org/extracellular_vesicle_markers

[30] Percie Du Sert N, Hurst V, Ahluwalia A, Alam S, Avey MT, Baker M, et al. The ARRIVE guidelines 2.0: updated guidelines for reporting animal research. BMJ Open Science. 2020;44. doi: 10.1136/bmjos-2020-100115 34095516 PMC7610906

[31] Foster JB, Zhao F, Wang X, Xu Z, Lin K, Askwith CC, et al. Pyridazine-derivatives Enhance Structural and Functional Plasticity of Tripartite Synapse Via Activation of Local Translation in Astrocytic Processes. Neuroscience. 2018;388: 224–238. doi: 10.1016/j.neuroscience.2018.07.028 30056115 PMC6667176

[32] DAVID: Functional Annotation Result Summary. In: DAVID Functional Annotation Tool [Internet]. [cited 29 Jan 2024]. https://david.ncifcrf.gov/summary.jsp

[33] Home | HUGO Gene Nomenclature Committee. In: DAVID Functional Annotation Tool [Internet]. [cited 29 Jan 2024]. https://genenames.org/

[34] Expression Heat Map. In: Heatmapper Expression [Internet]. www.heatmapper.ca/expression/

[35] Huang Y, Cheng L, Turchinovich A, Mahairaki V, Troncoso JC, Pletniková O, et al. Influence of species and processing parameters on recovery and content of brain tissue‐derived extracellular vesicles. J of Extracellular Vesicle. 2020;9: 1785746. doi: 10.1080/20013078.2020.1785746 32944174 PMC7480582

[36] Vella LJ, Scicluna BJ, Cheng L, Bawden EG, Masters CL, Ang C, et al. A rigorous method to enrich for exosomes from brain tissue. J of Extracellular Vesicle. 2017;6: 1348885. doi: 10.1080/20013078.2017.1348885 28804598 PMC5533148

[37] Veerman RE, Teeuwen L, Czarnewski P, Güclüler Akpinar G, Sandberg A, Cao X, et al. Molecular evaluation of five different isolation methods for extracellular vesicles reveals different clinical applicability and subcellular origin. J of Extracellular Vesicle. 2021;10: e12128. doi: 10.1002/jev2.12128 34322205 PMC8298890

[38] Karimi N, Cvjetkovic A, Jang SC, Crescitelli R, Hosseinpour Feizi MA, Nieuwland R, et al. Detailed analysis of the plasma extracellular vesicle proteome after separation from lipoproteins. Cell Mol Life Sci. 2018;75: 2873–2886. doi: 10.1007/s00018-018-2773-4 29441425 PMC6021463

[39] Gámez-Valero A, Monguió-Tortajada M, Carreras-Planella L, Franquesa M, Beyer K, Borràs FE. Size-Exclusion Chromatography-based isolation minimally alters Extracellular Vesicles’ characteristics compared to precipitating agents. Sci Rep. 2016;6: 33641. doi: 10.1038/srep33641 27640641 PMC5027519

[40] Kumar R, Tang Q, Müller SA, Gao P, Mahlstedt D, Zampagni S, et al. Fibroblast Growth Factor 2‐Mediated Regulation of Neuronal Exosome Release Depends on VAMP3/Cellubrevin in Hippocampal Neurons. Advanced Science. 2020;7: 1902372. doi: 10.1002/advs.201902372 32195080 PMC7080514

[41] Datta Chaudhuri A, Dasgheyb RM, DeVine LR, Bi H, Cole RN, Haughey NJ. Stimulus‐dependent modifications in astrocyte‐derived extracellular vesicle cargo regulate neuronal excitability. Glia. 2020;68: 128–144. doi: 10.1002/glia.23708 31469478

[42] Böing AN, Van Der Pol E, Grootemaat AE, Coumans FAW, Sturk A, Nieuwland R. Single‐step isolation of extracellular vesicles by size‐exclusion chromatography. J of Extracellular Vesicle. 2014;3: 23430. doi: 10.3402/jev.v3.23430 25279113 PMC4159761

[43] Colhoun HM, Otvos JD, Rubens MB, Taskinen MR, Underwood SR, Fuller JH. Lipoprotein Subclasses and Particle Sizes and Their Relationship With Coronary Artery Calcification in Men and Women With and Without Type 1 Diabetes. Diabetes. 2002;51: 1949–1956. doi: 10.2337/diabetes.51.6.1949 12031985

[44] Brunel A, Bégaud G, Auger C, Durand S, Battu S, Bessette B, et al. Autophagy and Extracellular Vesicles, Connected to rabGTPase Family, Support Aggressiveness in Cancer Stem Cells. Cells. 2021;10: 1330. doi: 10.3390/cells10061330 34072080 PMC8227744

[45] Blanc L, Vidal M. New insights into the function of Rab GTPases in the context of exosomal secretion. Small GTPases. 2018;9: 95–106. doi: 10.1080/21541248.2016.1264352 28135905 PMC5902209

[46] Van IJzendoorn SCD. Recycling endosomes. Journal of Cell Science. 2006;119: 1679–1681. doi: 10.1242/jcs.02948 16636069

[47] Hsu C, Morohashi Y, Yoshimura S, Manrique-Hoyos N, Jung S, Lauterbach MA, et al. Regulation of exosome secretion by Rab35 and its GTPase-activating proteins TBC1D10A—C. Journal of Cell Biology. 2010;189: 223–232. doi: 10.1083/jcb.200911018 20404108 PMC2856897

[48] Lischnig A, Bergqvist M, Ochiya T, Lässer C. Quantitative Proteomics Identifies Proteins Enriched in Large and Small Extracellular Vesicles. Molecular & Cellular Proteomics. 2022;21: 100273. doi: 10.1016/j.mcpro.2022.100273 35918030 PMC9486130

