# Supplementary figures and images for: Comparison of the protein composition of isolated extracellular vesicles from mouse brain and dissociated brain cell culture medium
Source: PLoS One. 2024 Nov 12;19(11):e0309716. doi: 10.1371/journal.pone.0309716 (PMC11556680; doi:10.1371/journal.pone.0309716)

**S1 Fig. EV Isolation Process.**

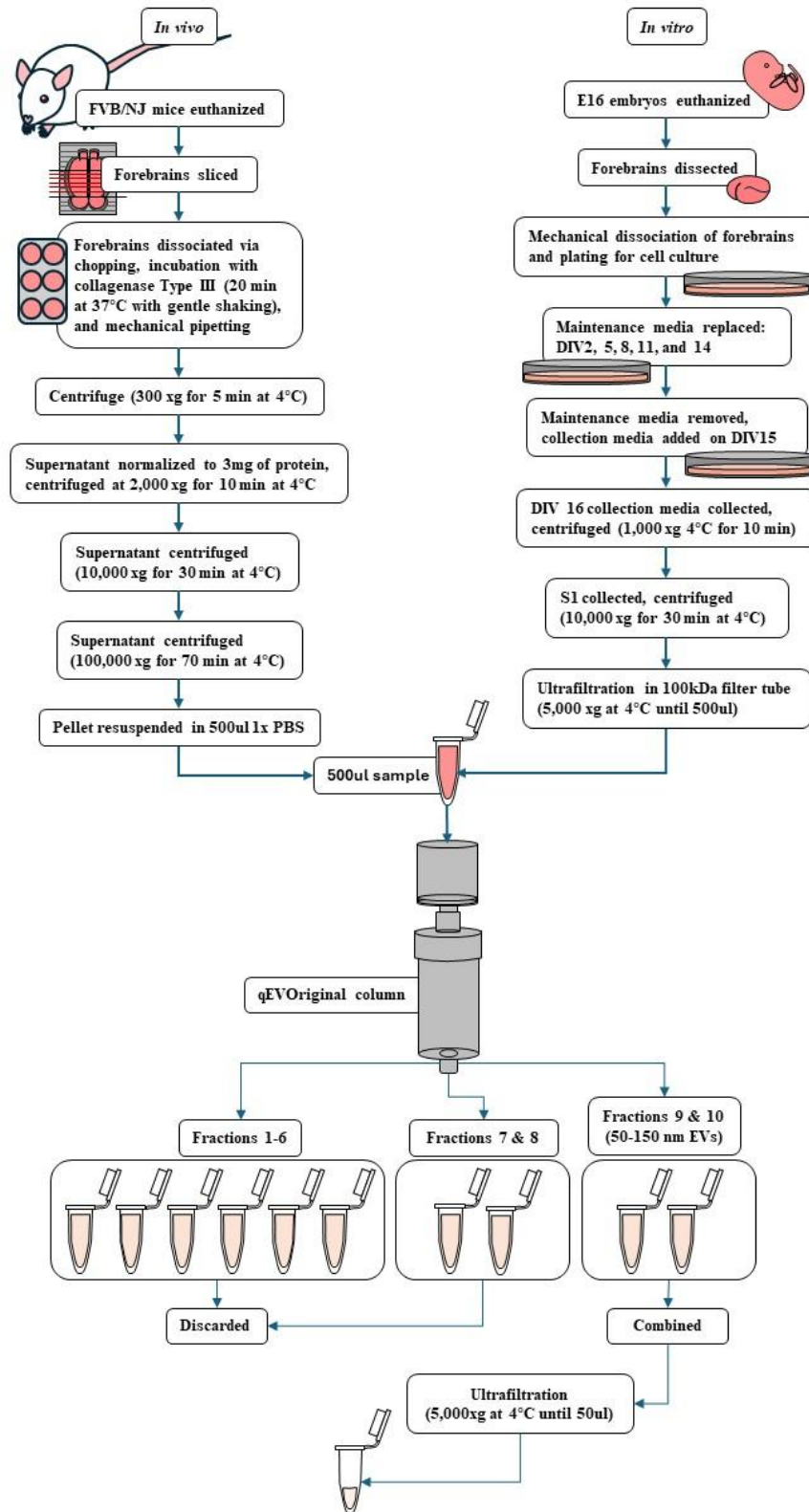

Supplement: S1 Fig — The figure gives a condensed pictorial outline of the EV collection and isolation methods from both in vivo and in vitro sources outlined in the Materials and Methods section of this manuscript. (PDF) [file pone.0309716.s001.pdf]

**S2 Fig. Western Blot Analysis of *in vivo* EV Isolation Via SEC Column and Ultracentrifugation.**

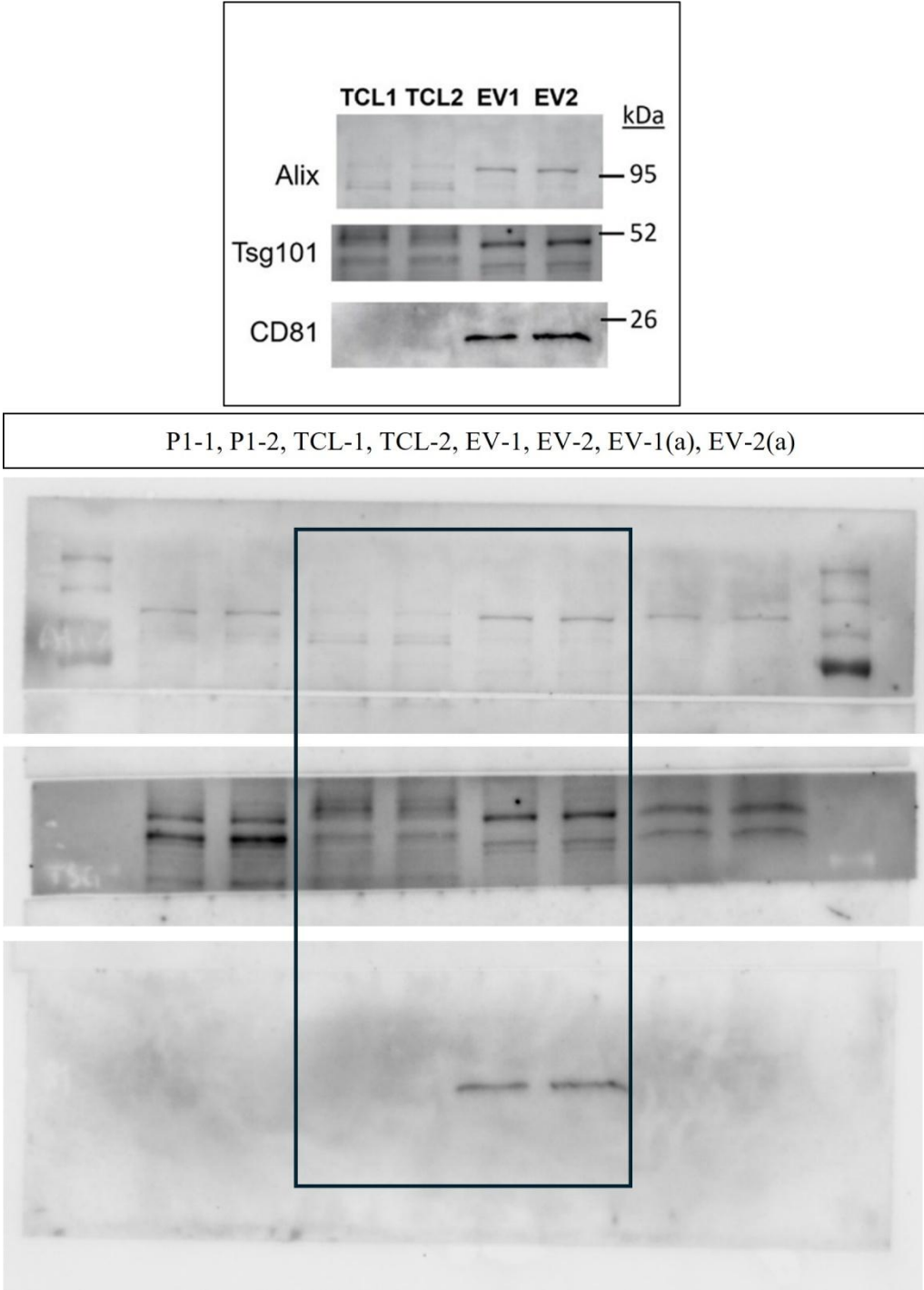

Supplement: S2 Fig — These results showcase Western Blot analysis of in vivo EV isolation via SEC column and ultracentrifugation. Normalized amounts of protein were resolved by SDS—PAGE (8% or 10% polyacrylamide gel) and transferred onto nitrocellulose membranes. Membranes were probed against antibodies of interest including: TSG101 (ThermoFisher, MA1-23296), CD81 (ThermoFisher, MA5-32333), and Alix (Cell signaling, 2171S). Subsequently, goat anti-rabbit IgG or anti-mouse IgG secondary antibodies (Bio-Rad; 1:5,000) conjugated to horseradish-peroxidase were incubated with the blot. For visualization of immunoreactive bands, either WesternBright or WesternBright Sirius (Advansta) enhanced chemiluminescence substrates were applied according to the manufacturer’s directions. Digital images of immunoblots were captured using the ChemiDoc Imaging System (Bio-Rad) and band intensities were analyzed using Image Lab (Bio-Rad). Samples are labeled by “sample type-number(addendum).” P1 indicates the pellet of the second centrifugation (2,000 x g for 10 min) post-homogenization and dissociation. Whereas, TCL in this case refers to the supernatant of the first centrifugation (300 x g for 5 min) post-homogenization and dissociation, as this low speed spin was used exclusively to remove excess tissue debris and undissociated tissue remnants. Samples labeled EV are in reference to samples that underwent the entire EV isolation process through ultracentrifugation and SEC column isolation. The addendum “(a)” indicates the EV samples that were processed by a trainee and did not contain enough signal to showcase CD81, potentially due to user error. Western Blot analysis is not shown for in vitro EV isolation due to weak signaling from the cell culture samples, but EV markers were confirmed via mass spectroscopy and comparison with the MISEV2023 recommendations categories and Vessiclepedia Top100 list of most often identified EV proteins as seen in Figs 4 and 5, respectively, in the main body of this manusc [file pone.0309716.s002.pdf]
